# Fluoroquinolone Derivatives in the Treatment of *Mycobacterium tuberculosis* Infection

**DOI:** 10.3390/ph15101213

**Published:** 2022-09-30

**Authors:** João Pedro Pais, Margarida Policarpo, David Pires, Ana Paula Francisco, Ana Margarida Madureira, Bernard Testa, Elsa Anes, Luís Constantino

**Affiliations:** 1 Research Institute for Medicines and Pharmaceutical Sciences (iMed.UL), Av. Prof. Gama Pinto, 1649-003 Lisboa, Portugal; 2 Faculty of Pharmacy, University of Lisbon, Av. Prof. Gama Pinto, 1649-003 Lisboa, Portugal; 3 University of Lausanne, 1015 Lausanne, Switzerland

**Keywords:** fluoroquinolones, esters, prodrugs, tuberculosis, mycobacteria

## Abstract

Tuberculosis (TB) is currently one of the leading causes of death due to infective agents, and the growing rate of multidrug-resistant tuberculosis (MDR TB) cases poses an emergent public health threat. Fluoroquinolones are commonly used in the treatment of both MDR TB and drug-sensitive tuberculosis patients who are intolerant to first-line antitubercular agents. Unfortunately, these drugs have mild side effects, relevant to the prolonged treatment regimens and diminished bioavailability due to binding of metal ions. Moreover, the resistance to fluoroquinolones is also on the rise, a characteristic of extensively drug-resistant TB (XDR TB). Here, we developed esters as prodrugs of the fluoroquinolones levofloxacin and ciprofloxacin, with long-chain fatty alcohols. Both the alcohols and the quinolone have previously shown antimycobacterial activity and the aim was to develop esters with improved lipophilicity and capable of delivering the free acid inside mycobacterial cells. The carboxylic acid group of fluoroquinolones is essential to the mode of action but is also responsible for many of its side effects and metal-chelating properties. The synthesis, stability in biological media, and antibacterial activity were evaluated, the latter not only against *Mycobacterium tuberculosis* but also against other clinically relevant bacterial species, since the parent compounds display a broad spectrum of activity. The biological results show a reduction in the antitubercular activity of the synthesized derivatives, probably due to deficient activation of the ester prodrug. Despite this, it was found that the derivatives exhibit bioactivity against other fluoroquinolone-resistant bacteria, indicating a different mode of action and suggesting that it may be worthwhile to research further modifications to the carboxylic acid group. This might lead to new compounds that are efficient against resistant strains. This idea that the compounds may act by a different mechanism of action was further supported by a brief computer investigation that demonstrated the potential lack of selectivity of the esters to the fluoroquinolone target.

## 1. Introduction

Tuberculosis (TB) remains one of the leading infectious causes of death globally, and a major concern for the World Health Organization (WHO). The coronavirus disease 2019 (COVID-19) pandemic has heavily impacted the progress towards TB milestones and targets, with the number of people dying from TB increasing in 2020 and a further global increase in TB incidence projected for 2022 and 2023 [1]. To support countries in responding to the challenges of TB and drug-resistant TB (DR-TB), including extensively drug-resistant TB (XDR-TB) and pre-XDR-TB, the WHO provides consolidated guidelines, with the latest being from June 2020 [2].

Fluoroquinolones (FQs) have been an invaluable weapon against TB and are used clinically in combinations for both first- and second-line treatments. Moreover, FQs comprise some of the most widely used antibacterial drugs, mainly due to their high potency and large spectrum of activity. Their mechanism of action relies in binding to one or both type II bacterial topoisomerase enzyme domains, DNA gyrase and topoisomerase IV, resulting in the inhibition of DNA replication and transcription [3]. In *Mycobacterium tuberculosis* (MTB), FQs have DNA gyrase as the sole target; hence, resistance to these compounds is primarily due to mutations in the gyrA and gyrB genes, responsible for encoding DNA gyrase [4]. A secondary mode of resistance, or at least of reduced susceptibility, are efflux systems, having been identified in multiple mycobacterial species, and some putative systems in MTB [5].

FQs remains essential to the treatment of TB, with new combination therapies recently showing the capacity to reduce the treatment time for TB cure to 4 months [6]. In order to fight antibiotic resistance, several strategies can be applied. One of such approaches is the synthesis of prodrugs of known antibiotics, since it may be able to mask activity and/or toxicity while improving the PD profile of the drug [7]. Ester prodrugs are widely used, and there are several examples of such methodologies potentially useful in promoting the crossing of the MT bacterial cell wall, one of the major hurdles of present therapeutic agents [8].

There are some examples in the literature of the development of fluoroquinolone esters and assessment of their antimicrobial properties, mainly of methyl and ethyl esters or an ester linkage to other complex molecules in diverse approaches [9,10,11,12,13,14]. Unfortunately, a general result of these efforts is an observed reduction in the antimicrobial activity for the esters, unless when challenging resistant strains.

Nonetheless, no extensive report of antimycobacterial activity was found for such derivatives, and an ester approach is specifically suited for the problem at hand, since MTB expresses diverse esterase enzymes due to their unique fatty acid metabolism [8]. The metabolization of host lipids as an essential source of nutrients indicates the existence of esterases capable of hydrolyzing long-chain fatty esters, a unique trait among the major pathogenic bacteria.

The present work aims to make use of this fact by developing prodrugs of relevant fluoroquinolones, namely, levofloxacin (LEV) and ciprofloxacin (CIP), where the carboxylic acid moieties present are masked by esterification with medium- to long-chain fatty alcohols, to facilitate the access of these FQs to the intracellular cytosolic milieu of MTB bacilli. We hypothesize that these proposed derivatives would be internalized and activated by the MTB hydrolases, resulting not only in active FQ compounds but also in the corresponding fatty alcohols, which previous studies have found do possess antitubercular activity per se [15].

## 2. Results

### 2.1. Synthesis

**Figure 1:**
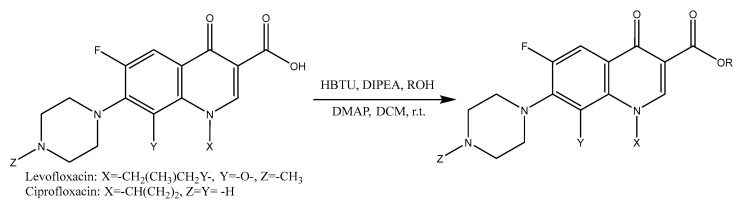
General synthetic scheme for fluoroquinolone esters.

The preparation of fluoroquinolone esters was performed as described in Figure 1, using HBTU as a coupling reagent. As other amide coupling reagents, the classical HBTU methodology is commonly applied for the formation of peptide bonds [16,17]; however, reports show that it can be adapted to the formation of esters with good yields [18]. Derivatives of two fluoroquinolones were prepared, and while LEV was promptly reacted with the desired alcohol (Figure 1), CIP required extra protection–deprotection steps, due to the secondary amine residue.

Several primary linear alcohols were used in the synthesis of a series of derivatives, ranging from hexanol to hexyldecanol, with the shorter chain lengths resulting in higher synthetic yields. This series of derivatives provides a range from moderate to highly lipophilic potential ester prodrugs (Table 1).

### 2.2. Susceptibility to Hydrolysis

The fluoroquinolone prodrugs synthesized in this study, being esters, are structures susceptible to hydrolysis; hence, their chemical and enzymatic stability was assessed, using the described methodology. With the HPLC analysis, it was possible to observe the disappearance of the ester and the quantitative correspondent emergence of the respective acid, and the results obtained are presented in Table 1.

The hydrolysis results show low levels of degradation, both chemical and plasmatic, and the chemical stability of these derivatives does not impact the degradation observed in plasma. The fact that low degradation is observed, even in the presence of plasma esterases, is a desirable prodrug characteristic since the ester is expected to exhibit increased absorption and cellular integration when compared to the main pharmacophore. Additionally, considering the values for Compounds 1–6 for PBS and Compounds 2–7 for plasma, an inverse correlation can be observed (y = −1.24x + 8.42, *r^2^* = 0.905 and y= −5.65x + 40.4, *r^2^* = 0.959, respectively) between the lipophilicity of the derivatives and their susceptibility to hydrolysis.

Following the previous results, the next essential step to the prodrug approach is the activation of the prodrug in the target site; in this case, their activation by mycobacteria. In order to assess this, the derivatives were incubated with a mycobacterial homogenate solution, previously calibrated to achieve a hydrolysis rate of ethyl benzoate in accordance to the literature [20]. From the exposure of the derivatives to the mycobacterial homogenate, for 24 h and further extended to 48 h, no hydrolysis was observed via HPLC.

### 2.3. Antitubercular Activity

The derivatives synthesized were tested against the reference strain *Mycobacterium tuberculosis* H37Rv, and the results obtained are presented in Table 2. Both the minimal inhibitory concentration (MIC) as well as minimal bactericidal concentration (MBC) are presented.

Additionally, since the antibacterial profile is modified, we assessed the cytotoxicity of the new derivatives. The human macrophage-derivative THP-1 cell line was challenged with the synthesized derivatives and the results obtained are presented in Table 2, as the lethal concentration for 50% of the cellular culture (LC50).

Interestingly, the results obtained show an inverse correlation between the antimycobacterial activity and cytotoxicity. Despite the small sample of derivatives, the chain length of the ester shows an inverse correlation with the antitubercular activity, with derivatives with increasing chain lengths showing diminishing activity, and a direct correlation to the cytotoxicity, with increasing chain lengths showing increased cytotoxicity.

Compound **10** does not follow the trend described, showing low toxicity and antitubercular activity, but being the most lipophilic of the tested compounds, its low biological activity can probably be attributed to its very low solubility in the experimental medium.

### 2.4. Antibacterial Activity

Fluoroquinolones are broad-spectrum antibiotics; hence, it is relevant to study the effect of esterification not only in the antibiotic activity but also in the selectivity. For this reason, several clinically relevant bacterial strains were selected, both Gram positive (Gram+) and Gram negative (Gram−), and challenged with the synthesized derivates. Selected results obtained are presented in Table 3 and the complete set of results in Table S1.

As expected, in concordance with the available literature, the MIC values obtained were increased for the ester derivatives when compared to the corresponding parent compounds, levofloxacin and ciprofloxacin. Nonetheless, several other conclusions can be drawn from the resulting data.

Evaluating the results obtained, it is clear that the bioactivity for Gram− and Gram+ bacteria show distinct trends. Similar to the observed against mycobacteria, the MIC values for Gram– bacteria tend to increase with increasing ester chain length. On the other hand, the MIC values for Gram+ bacteria show no direct correlation between the chain length and the antibacterial activity; instead, the emergence of an optimal chain length is apparent. Compounds **4** and **5** are generally the most active compounds, comprising aliphatic chains of **9** and **10** carbon atoms, respectively, and this could be defined as the optimal chain length for this type of derivative. Interestingly, the aforementioned compounds retain the same antibacterial activity against drug-resistant strains (MRSA ATCC 43866), even when challenging a fluoroquinolone-resistant bacterial strain (VISA CIP 106760).

### 2.5. Docking Studies

Since the synthesized compounds have shown to be stable in biological media and still presented antibacterial activity, we decided to explore if the mode of action could be retained in the ester derivatives. For that, a small computational study was performed using a crystallographic structure of MTB DNA gyrase obtained from a protein data bank (PDB: 5BTG). Protein and ligand preparations were performed using AutoDock Tools software suite and the docking was performed using Autodock 4.2.6 [21]. This study aimed at comparing the affinity of levofloxacin and its hexyl and nonyl esters to the known binding pockets in the DNA gyrase; hence, the first step is to validate the docking process by re-docking the co-crystalized ligand, levofloxacin in this case.

**Figure 2:**
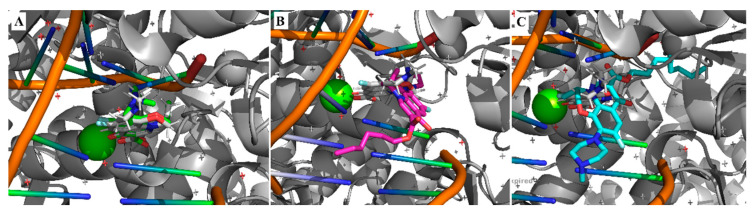
Top pose obtained from the docking of levofloxacin (**A**), hexyl ester of LEV (**B**) and nonyl ester of LEV (**C**).

Great concordance between the crystallographic pose of levofloxacin and the docking result could be observed for levofloxacin (Figure 2A), with the RMSD value of 0.48 Å validating the docking approach. Meanwhile, the best poses for Compounds **1** and **4** (Figure 2B,C) showed very poor similarities, with RMSDs of 7.72 Å and 5.74 Å, respectively.

**Figure 3:**
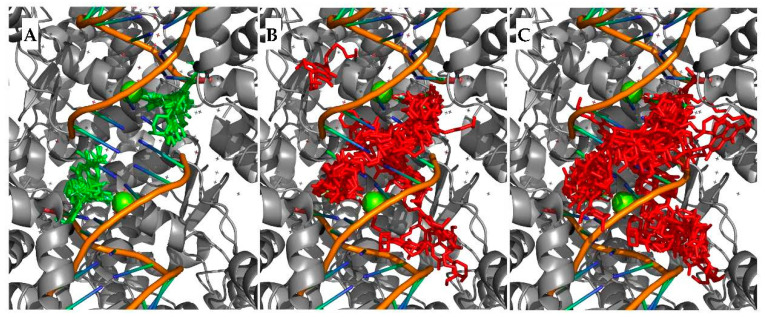
Top 50 poses obtained from the docking of levofloxacin (**A**), hexyl ester of LEV (**B**), and nonyl ester of LEV (**C**).

Furthermore, since from the crystallographic structures available for levofloxacin with Mtb topoisomerase II (PDB: 5BTG and 5BTI) it can be observed that there are two binding sites, the entire dispersion of the poses obtained was studied, as a better proxy for loss of selectivity. While the docking of LEV showed selectivity only to these positions (Figure 3A), with an average root mean standard deviation (RMSD) of 0.69 Å for the top 25 poses, the docking of the esters showed significantly different results, with no selectivity to the binding pockets being observed (Figure 3B and 3C); for comparison, the hexyl and nonyl esters showed average RMSDs of 6.50 Å and 7.06 Å, respectively.

## 3. Discussion

Prodrugs can offer a wide variety of advantages in multiple applications, but it all rests on the principle that the derivatives are absorbed, transported, and delivered in its inactivated form, and once in the target of interest, are successfully activated.

The fluoroquinolone ester derivatives here presented were synthesized with such an approach in mind, aiming at increased activity on *Mycobacterium tuberculosis* due to enhanced absorption, resulting from the higher lipophilicity of the compounds.

Unfortunately, the results obtained showed that such an approach is not ideal, probably due the high resistance of such esters to hydrolysis, hindering their activation in the target organism. This work showed that such derivatives are stable not only chemically but also in the presence of plasma and mycobacterial esterases, showing very low levels of degradation and only for longer time frames. Such stability in the presence of bacterial esterases, specially from a bacterial species with specialized fatty lipid metabolism, indicate that any bioactivity observed for the synthesized derivatives should be due to the complete molecular entity and not to the “activated” ciprofloxacin or levofloxacin as previously expected. This result contrasts with the easy activation by mycobacterial esterases of other prodrugs that we obtained in previous studies [22,23].

If this result is to be extrapolated to the fluoroquinolone esters presented in the literature [9,10,11,12,13,14], it could offer an explanation to the generally reduced antibacterial activity observed for such types of prodrugs and/or codrugs. Likewise, the results obtained from the antitubercular and antimicrobial assays show a diminished activity when compared to the parent compounds; nonetheless, there is interesting activity overall.

By comparing the obtained values for MIC and MBC, it is apparent that the bioactivity observed is bactericidal in nature, consistent with the mechanism of action (MoA) of fluoroquinolones. This evaluation becomes increasingly important when realizing the direct correlation between the susceptibility of the compounds towards hydrolysis and their antitubercular bactericidal action, bringing to question if the antitubercular activity is due to the action of the fluoroquinolone esters or the small fraction of ester that is hydrolyzed during the incubation period with the compounds under study (10–15 days), liberating a small percentage of free fluoroquinolone and fatty alcohol.

A similar observation was made for the bioactivity in Gram-negative bacteria, also showing a similar correlation.

Conversely, the antimicrobial activity observed against Gram-positive bacteria shows a new pattern of activity, with an ester chain length of nine and ten carbon atoms being the most active (Compounds **4** and **5**). In this case, the existence of an optimal chain-length breaks the correlation between activity and hydrolysis, supporting the hypothesis that such compounds are showing biological activity per se. Additionally, by comparing the activity of these compounds against FQ-susceptible and FQ-resistant strains, the fact that both the MIC and MBC values remained largely unaltered is an interesting indicator that such derivatives are inhibiting bacterial growth via a diverse MoA of their parent compound; in this case, levofloxacin.

Additionally, the small computational study performed also found that while the docking of levofloxacin demonstrated affinity only to the binding sites present in the crystallographic structure, our derivatives were dispersed over the grid-box area, with no clear affinity to these sites. This is a strong indication of potential loss of selectivity to this target, highlighting the possibility of such derivatives possessing a diverse mode of action for the antimycobacterial activity, when compared to the parent compound.

In conclusion, fluoroquinolone esters have some drawbacks as a prodrug for the treatment of tuberculosis, but as novel molecular entities, they also exhibit interesting antimicrobial activity and a potential new mode of action, suggesting that changes to the carboxylic acid group may be worth further investigation in order to develop new drugs that are effective against resistant strains.

## 4. Materials and Methods

Materials. Balanced salt solution, phosphate-buffered saline (PBS), Dulbecco’s modified Eagle’s medium (DMEM), and L-glutamine were purchased from Invitrogen. Hexanol, heptanol, octanol, nonanol, decanol, undecanol, dodecanol, tridecanol, tetradecanol, hexadecanol, levofloxacin, and ciprofloxacin were purchased from Sigma-Aldrich Quimica SA. Middlebrook 7H10 agar was purchased from Difco. Microwell tissue culture plates were purchased from Nunc. All esters presented were synthesized according to the general method described below. Compounds were prepared in stock solutions of 40 mg/mL in dimethyl sulfoxide (DMSO—AppliChem Panreac). Isoniazid (Sigma-Aldrich, St. Louis, MO, USA) is a first-line antibiotic against tuberculosis and was used as a positive control for *M. tuberculosis* killing. Bacteria broth culture medium Middlebrook 7H9 and solid culture medium Middlebrook 7H10 were purchased from Difco and were supplemented with OADC (oleic acid, albumin, dextrose, catalase—Sigma-Aldrich) and tyloxapol (Sigma-Aldrich).

Bacterial strains and culture conditions. *Escherichia coli* ATCC 8739, *Salmonella typhimurium* ATCC 13311, *Enterococcus faecalis* ATCC 11420; *Enterococcus faecalis* ATCC 51299, Vancomycin-resistant *Staphylococcus aureus* (VISA) CIP 106760; *Staphylococcus aureus* subsp. *aureus* Rosenbach ATCC 6538; *Staphylococcus aureus* subsp. *aureus* Rosenbach ATCC 43866; *Staphylococcus aureus* CIP 106414, ATCC 700699—these were cultivated in solid media, incubated at 37 °C for 24 to 48 h, and colonies were selected and suspended in Muller–Hinton (MH) broth, for MIC determination. *Mycobacterium tuberculosis* H37Rv (ATCC 27294) were cultivated in Middlebrook 7H9 medium supplemented with OADC and 0.05 % tyloxapol (Sigma-Aldrich) and incubated at 37 °C until an exponential growth phase was achieved. The THP-1 macrophage cell line was used for the LC50 determination.

NMR analysis. *^1^H*-NMR and *^13^C*-NMR spectra were recorded on a Bruker MS-400 (at 400 MHz for *^1^H* and 75 MHz for *^13^C*) instrument, with tetramethylsilane as internal reference and in chloroform-*d* as solvent; chemical shifts are reported in ppm. *^1^H*- and *^13^C*-NMR signals were obtained at room temperature.

HRMS analysis. High-resolution mass spectra in the ESI-positive mode were obtained on a QqTOF Impact II TM mass spectrometer (Bruker Daltonics) operating in the high-resolution mode. Samples were analyzed by flow-injection analysis (FIA) using an isocratic gradient 30 A:70 B of 0.1% formic acid in water (A) and in acetonitrile (B), at a flow rate of 10 µL/min over 15 min. Internal calibration was achieved with a solution of ammonium formate 10 mM introduced to the ion source via a 20 µL loop at the beginning of each analysis, using a six-port valve. The full scan mass spectra were acquired over a mass range of 100–1000 m/z at a spectra rate of 1 Hz. Data acquisition and processing were performed using Data Analysis 4.2 software.

FT-IR analysis. FT-IR spectra were obtained on an IRAffinity-1 Fourier Transform Infrared (FTIR) spectrophotometer (Shimadzu) using KBr pellets.

HPLC analysis. Two HPLC systems were used. The buffer and plasma stability studies were performed in an HPLC system with a photodiode detector L-3000 Photo Diode Array Detector, Merck-Hitachi L-6000 pump, Merck-Hitachi D-2500 integrator and Merck RP-8 column. Activation studies were performed in an HPLC system with a UV detector Merck-Hitachi UV-L7400, Merck Hitachi L-7100 pump, an auto sampler Merck-Hitachi AS 2000, a Merck-Hitachi D-2500 integrator, and Merck RP-8 column. The eluant was a mixture of acetonitrile (80%) and aqueous phosphate buffer with 0.5% of KH_2_PO_4_/H_3_PO_4_ 0.0025 M (20%). The flow rate was always 1 mL/min and the wavelength was set at 295 nm. All quantifications were evaluated using calibration curves from stock solutions.

Plasma stability. Pooled human plasma (640 µL), pH 7.4 phosphate-buffered saline (160 µL), and 16 µL of a 2.5 × 10^−2^ M stock solution of the prodrug in ACN were mixed in a 2 mL vial. The suspensions were incubated at 37 °C and 50 µL aliquots were removed, mixed with 450 µL of ACN, and centrifuged. The supernatant was removed and analyzed by HPLC—the remaining substrate and the correspondent acid measured.

Buffer stability. A 1640 µL pH 7.4 phosphate buffer, 320 µL of ACN, and 40 µL of a 2.5 × 10^−2^ M stock solution of the prodrug in ACN were mixed in a 2 mL vial. The solution was incubated at 37 °C and 50 µL aliquots were removed, mixed with 450 µL of ACN. The solution was analyzed by HPLC—the remaining substrate and the correspondent acid were both measured.

Hydrolysis in *M. smegmatis* homogenate. *M. smegmatis* homogenate was prepared using the technique described in previous work [20]. The incubation of the homogenate with the prodrug was performed at 37 °C in a total volume of 1500 µL. The concentration of substrate in the incubation media was 5 × 10^4^ M, and the protein concentration was 0.38 mg/mL. Dilutions were performed using PBS. At predetermined time points, aliquots of 50 µL were taken into vials containing 225 µL of acetonitrile and 225 µL of a 1% ZnCl_2_ aqueous solution. The suspension then was agitated in a vortex and centrifuged for 15 min. The supernatant was injected into the HPLC for quantification of the FQ ester and FQ.

MIC and MBC determination. The MIC of each compound was evaluated using the microdilution method of serial dilutions in 96-well plates, testing drug concentrations ranging from 256 to 4 µg mL^−1^ for all bacterial strains tested.

For mycobacteria: These essays were performed in Middlebrook 7H9 medium supplemented with OADC containing an adjusted concentration of mycobacteria corresponding to approximately 10^5^ CFU of organisms/mL. Incubation was performed for 10 days at 37 °C. Isoniazid, DMSO, and a well with no drug were used as controls. The MIC corresponds to the well concentration where no visible turbidity was observed. The MBC of compounds was evaluated by plating serial dilutions of each well into Middlebrook 7H9 solid media. The MBC corresponds to the concentration used in the respective well where no colony-forming units (CFU) were observed.

For other bacteria: These essays were performed in MH broth containing an adjusted concentration of bacteria corresponding to approximately 10^6^ CFU of organisms/mL. Incubation was performed for 24 h at 37 °C. DMSO and a well with no drug were used as controls. The MIC corresponds to the well concentration where no visible turbidity was observed. The MBC of compounds evaluated by plating an aliquot of each well into solid media (Muller–Hinton agar) and incubated for 24 h–48 h. The MBC corresponds to the concentration used in the respective well where no CFUs were observed.

Cytotoxicity assay. The Human monocytic cell line THP-1 (ATCC TIB202) was used to determine the cytotoxicity effect of the compounds. The cells were grown in RPMI 1640 (Gibco) supplemented with 10 % fetal bovine serum (FBS; Gibco), 10 mM HEPES (Gibco), 1 mM sodium pyruvate, and maintained at 37 °C with 5% CO_2_. Differentiation of THP-1 monocytes into macrophages was induced for 48 h with 20 nM phorbol 12-myristate 13-acetate (PMA) following a 24 h resting period without PMA. Differentiated macrophages in 96-well plates, 5 × 10^4^ cells per well, were treated with serial dilutions of the compounds. After three days of treatment, cell viability was determined using PrestoBlue (Invitrogen) following the manufacturer’s indications. Briefly, the cells were washed with PBS and incubated with PrestoBlue 10% (*v/v*) in cell culture medium. After 4 h of incubation, the fluorescence of each well was measured in a Tecan M200 spectrophotometer (Em: 560 nm/Ex: 590 nm). Viability was calculated relatively to non-treated cells. Cells treated with DMSO solvent at the same proportions were used as a control. Puromycin was used as a positive control for cell death.

Docking protocol. The docking studies were performed by using the AutoDock docking software version 4.2.6 [21]. The DNA gyrase structure was obtained from the RCSB protein data base (PDB ID 5BTG). Using AutoDock tools 1.5.7, the hydrogens and Kollman united atom type chargers were added. The AutoGrid program was used to generate the affinity (grid) maps of 80 × 80 × 80 Å grid points and 0.5 Å spacing, in order to contain both binding spots observed in the crystallographic structure.

Fluoroquinolone ester prodrugs synthesis—general procedure:

To a solution of the desired fluoroquinolone (0.2 mmol) in dichloromethane (DCM), HBTU (2.5 eq, 0.5 mmol) and a catalytic amount of DMAP (0.1 eq, 0.02 mmol) were added. The solution was placed under agitation, in a nitrogen atmosphere, at room temperature. After 15 min, a solution of the desired alcohol (2.5 eq, 0.5 mmol) and DIPEA (5 eq, 1 mmol) in DCM was slowly added. The reaction was followed by TLC (eluent: DCM:MeOH, 9.5:0.5) and when finished, DCM was added to the reactional mixture, and it was washed successively with a 10% bicarbonate solution (2×), a 5% citric acid solution (2×), and brine. The organic solution was subsequently dried, and the solvent evaporated. The residue was purified by column chromatography (silica gel 60) using a gradient eluent (10:0–9.5:0.5–DCM:MeOH).

Ciprofloxacin protection procedure:

To an aqueous solution of NaOH, ciprofloxacin hydrochloride ((1-cyclopropyl-6-fluoro-4-oxo-7-(piperazin-1-yl)-1,4-dihydroquinoline-3-carboxylic acid hydrochloride) and BOC_2_O were added consecutively. The reactional mixture was left at room temperature under magnetic stirring until completion, as observed by TLC. Once the reaction was completed, the solution was extracted with Et2O. Then, the aqueous layer was acidified to pH = 4–5 and then extracted with DCM, and this organic phase was dried over anhydrous Na_2_SO_4_ and evaporated under reduced pressure to give the Boc-protected ciprofloxacin as a white solid.

Ciprofloxacin deprotection procedure:

The ciprofloxacin ester was stirred in trifluoroacetic acid (TFA), and after the completion of the reaction, the product was precipitated with diethyl ether, filtered, washed, and further purified by column chromatography (silica gel 60) using a gradient eluent (10:0–9.5:0.5–DCM:MeOH).

Products:

Hexyl (S)-9-fluoro-2,3-dihydro-3-methyl-10-(4-methylpiperazin-1-yl)-7-oxo-7H-pyrido [1,2,3-de]-1,4-benzoxazine-6-carboxylate (**1**)

White solid; Yield 88%; m.p.:195–197 °C; ^1^H NMR (300 MHz, CDCl_3_), δ (ppm): 8.46 (s, 1H); 7.88 (d, J = 12.9 Hz, 1H); 7.31 (d, J = 6.9 Hz, 1H); 4.15 (t, J = 6.9 Hz, 2H); 3.41 (d, J = 3.8 Hz, 5H); 3.32 (d, J = 3.5 Hz, 4H); 3.22 (m, 1H); 1.63 (m, 2H); 1.28–1.20 (m, 8H); 1.00 (d, J = 3.0 Hz, 2H); 0.77 (t, J = 6.9 Hz, 3H). ^13^C NMR (75 MHz, CDCl_3_), δ (ppm): 173.90; 164.96; 155.01; 148.38; 143.39; 138.10; 123.64; 109.78; 106.08; 106.01; 64.99; 46.75; 43.29; 34.90; 31.33; 28.52; 25.54; 22.40; 13.73; 7.98. HRMS (ES+) *m/z*: 446.2456 (M + 1)^+^. FT-IR (ν cm^−1^): 2957; 2928; 2855; 2793; 1717; 1701; 1616; 1583; 1475; 1448; 1319; 1294; 1244; 1092; 798.

Heptyl (S)-9-fluoro-2,3-dihydro-3-methyl-10-(4-methylpiperazin-1-yl)-7-oxo-7H-pyrido [1,2,3-de]-1,4-benzoxazine-6-carboxylate (**2**)

White solid; Yield 94%; m.p.:187–189 °C; ^1^H NMR (300 MHz, CDCl_3_), δ (ppm): 8.25 (s, 1H); 7.59 (d, J = 12.6 Hz, 1H); 4.38–4.22 (m, 5H); 3.36 (d, J = 4.5 Hz, 4H); 2.59 (t, J = 4.8 Hz, 4H); 2.39 (s, 3H); 1.85–1.68 (m, 2H); 1.53 (d, J = 6.9 Hz, 3H); 1.48–1.24 (m, 8H), 0.88 (t, J = 6.8 Hz, 3H). ^13^C NMR (75 MHz, CDCl_3_), δ (ppm): 172.92; 165.72; 157.33; 154.09; 145.12; 139.62; 131.55; 123.79; 109.88; 105.65; 68.15; 65.08; 55.68; 54.73; 50.39; 46.28; 31.72; 28.89; 28.78; 25.95; 22.60; 18.18; 14.06. HRMS (ES+) *m/z*: 460.2617 (M + 1)^+^. FT-IR (ν cm^−1^): 2955; 2928; 2855; 2793; 1717; 1686; 1618; 1584; 1548; 1477; 1448; 1435; 1412; 1389; 1375; 1346; 1319; 1292; 1244; 1204; 1175; 1155; 1092; 1072; 841; 798.

Octyl (S)-9-fluoro-2,3-dihydro-3-methyl-10-(4-methylpiperazin-1-yl)-7-oxo-7H-pyrido [1,2,3-de]-1,4-benzoxazine-6-carboxylate (**3**)

White solid; Yield 24%; m.p.:186–187 °C; ^1^H NMR (300 MHz, CDCl_3_), δ (ppm): 8.23 (s, 1H); 7.59 (d, J = 12.6 Hz, 1H); 4.43–4.20 (m, 5H); 3.35 (d, J = 5.1 Hz, 4H); 2.57 (t, J = 4.8 Hz, 4H); 2.38 (s, 3H); 1.85–1.68 (m, 2H); 1.53 (d, J = 6.6 Hz, 3H); 1.45–1.16 (m, 10H), 0.87 (t, J = 6.8 Hz, 3H). ^13^C NMR (75 MHz, CDCl3), δ (ppm): 172.95; 165.89; 157.48; 154.21; 145.20; 139.72; 131.70; 123.82; 110.12; 105.96; 68.27; 65.22; 55.80; 54.83; 50.55; 46.42; 31.95; 29.43; 29.32; 28.93; 26.14; 22.77; 18.32; 14.21. HRMS (ES+) *m/z*: 474.2769 (M + 1)^+^. FT-IR (ν cm^−1^): 2953; 2920; 2851; 1718; 1612; 1578; 1508; 1481; 1412; 1318; 1242; 1155; 1090; 800.

Nonyl (S)-9-fluoro-2,3-dihydro-3-methyl-10-(4-methylpiperazin-1-yl)-7-oxo-7H-pyrido [1,2,3-de]-1,4-benzoxazine-6-carboxylate (**4**)

White solid; Yield 21%; m.p.:109–111 °C; ^1^H NMR (300 MHz, CDCl_3_), δ (ppm): 8.33 (s, 1H); 7.64 (d, J = 12.3 Hz, 1H); 4.39–4.23 (m, 5H); 3.48 (d, J = 3.6 Hz, 4H) 2.90 (s, 4H); 2.60 (s, 3H); 1.81–1.72 (m, 2H); 1.55 (d, J = 6.3 Hz, 3H); 1.42–1.18 (m, 12H); 0.87 (t, J = 6.6 Hz, 3H). ^13^C NMR (75 MHz, CDCl_3_), δ (ppm): 173.42; 165.70; 157.46; 154.19; 145.66; 140.12; 130.90; 124.07; 109.83; 105.90; 68.45; 65.32; 55.48; 55.02; 49.50; 45.64; 32.01; 29.63; 29.48; 28.93; 28.89; 26.14; 22.80; 18.28; 14.23. HRMS (ES+) *m/z*: 488.2931 (M + 1)^+^. FT-IR (ν cm^−1^): 2957; 2928; 2855; 1718; 1701; 1618; 1560; 1508; 1477; 1420; 1319; 1250; 1094; 845; 802; 557.

Decyl (S)-9-fluoro-2,3-dihydro-3-methyl-10-(4-methylpiperazin-1-yl)-7-oxo-7H-pyrido [1,2,3-de]-1,4-benzoxazine-6-carboxylate (**5**)

White solid; Yield 19%; m.p.:176–178 °C; ^1^H NMR (300 MHz, CDCl_3_), δ (ppm): 8.23 (s, 1H); 7.59 (d, J = 12.6 Hz, 1H); 4.45–4.18 (m, 5H); 3.33 (d.a., J = 4.8 Hz, 4H); 2.51 (t, J = 4.7 Hz, 4H); 2.31 (s, 3H); 1.82–1.70 (m, 2H); 1.53 (d, J= 6.6 Hz, 3H); 1.43–1.18 (m, 14H); 0.86 (t, J = 6.8 Hz, 3H). ^13^C NMR (75 MHz, CDCl_3_), δ (ppm): 172.92; 165.91; 157.48; 154.21; 145.20; 139.72; 131.71; 124.07; 110.12; 105.65; 68.27; 65.21; 55.83; 54.82; 50.60; 46.45; 32.02; 29.70; 29.67; 29.48; 29.44; 28.93; 26.14; 22.80; 18.32; 14.23. HRMS (ES+) *m/z*: 502.3075 (M + 1)^+^. FT-IR (ν cm^−1^): 2955; 2926; 2855; 2793; 1718; 1655; 1618; 1584; 1545; 1476; 1420; 1292; 1244; 1175; 1092; 1009; 798.

Undecyl (S)-9-fluoro-2,3-dihydro-3-methyl-10-(4-methylpiperazin-1-yl)-7-oxo-7H-pyrido [1,2,3-de]-1,4-benzoxazine-6-carboxylate (**6**)

Yellow solid; Yield 31%; m.p.:133–135 °C; ^1^H NMR (300 MHz, CDCl_3_), δ (ppm): 8.29 (s, 1H); 7.63 (d, J = 13.2 Hz, 1H); 4.43–4.18 (m, 5H); 3.46 (m, 4H); 2.67 (s, 4H); 2.43 (s, 3H); 1.81–1.67 (m, 2H); 1.51 (d, J = 6.6 Hz, 3H); 1.34–1.15 (m, 16H); 0.83 (t, J = 6.6 Hz, 3H). ^13^C NMR (75 MHz, CDCl_3_), δ (ppm):,42; 165.81; 157.43; 154.15; 145.43; 139.86; 131.41; 123.95; 109.86; 105.57; 68.30; 65.32; 55.46; 54.92; 50.34; 45.82; 31.95; 29.74; 29.67; 29.59; 29.39; 28.78; 26.04; 22.72; 18.19; 14.12. HRMS (ES+) *m/z*: 516.3238 (M + 1)^+^. FT-IR (ν cm^−1^): 2949; 2920; 2853; 1720; 1701; 1670; 1611; 1578; 1549; 1476; 1448; 1406; 1387; 1342; 1315; 1292; 1242; 1092; 1072; 800.

Dodecyl (S)-9-fluoro-2,3-dihydro-3-methyl-10-(4-methylpiperazin-1-yl)-7-oxo-7H-pyrido [1,2,3-de]-1,4-benzoxazine-6-carboxylate (**7**)

White solid; Yield 31%; m.p.:175–176 °C; ^1^H NMR (300 MHz, CDCl_3_), δ (ppm): 8.23 (s, 1H); 7.60 (d, J = 12.3 Hz, 1H); 4.41–4.22 (m, 5H); 3.34 (d, J = 4.5 Hz, 4H); 2.55 (t, J = 4.7 Hz, 4H); 2.36 (s, 3H); 1.82–1.70 (m, 2H); 1.54 (d, J = 6.6 Hz, 3H); 1.38–1.17 (m, 18H) 0.87 (t, J = 6.6 Hz, 3H). ^13^C NMR (75 MHz, CDCl_3_), δ (ppm): 172.95; 165.96; 157.52; 154.27; 145.21; 139.69; 131.79; 123.79; 110.24; 105.86; 68.27; 65.23; 55.86; 54.82; 50.68; 46.51; 32.05; 29.79; 29.76; 29.67; 29.48; 28.94; 26.15; 22.81; 18.32; 14.24. HRMS (ES+) *m/z*: 530.3401 (M + 1)^+^. FT-IR (ν cm^−1^): 2918; 2851; 1720; 1618; 1578; 1479; 1448; 1410; 1340; 1317; 1290; 1242; 1173; 1157; 1090; 1074; 800.

Tridecyl (S)-9-fluoro-2,3-dihydro-3-methyl-10-(4-methylpiperazin-1-yl)-7-oxo-7H-pyrido [1,2,3-de]-1,4-benzoxazine-6-carboxylate (**8**)

Yellow solid; Yield 43%; m.p.:124–126 °C; ^1^H NMR (300 MHz, CDCl_3_), δ (ppm): 8.39 (s, 1H); 7.56 (d, J = 12.6 Hz, 1H); 4.54–4.12 (m, 5H); 3.38 (d, J = 4.5 Hz, 4H); 2.68 (s, 4H) 2.45 (s, 3H); 1.81–1.69 (m, 2H); 1.47 (d, J = 6.6 Hz, 3H); 1.33–1.15 (m, 20H); 0.87 (t, J = 6.8 Hz, 3H). ^13^C NMR (75 MHz, CDCl_3_), δ (ppm): 174.41; 165.62; 157.54; 154.26; 145.91; 139.94; 131.72; 124.03; 108.66; 105.59; 68.35; 65.39; 55.48; 55.24; 50.27; 45.80; 31.96; 29.74; 29.72; 29.69; 29.62; 29.39; 28.64; 26.04; 22.71; 18.13; 14.10. HRMS (ES+) *m/z*: 544.3512 (M + 1)^+^. FT-IR (ν cm^−1^): 2953; 2918; 2851; 1718; 1612; 1578; 1551; 1477; 1410; 1340; 1319; 1292; 1242; 1173; 1155; 1090; 845; 800.

Tetradecyl (S)-9-fluoro-2,3-dihydro-3-methyl-10-(4-methylpiperazin-1-yl)-7-oxo-7H-pyrido [1,2,3-de]-1,4-benzoxazine-6-carboxylate (**9**)

Yellow solid; Yield 20%; m.p.:165–166 °C; ^1^H NMR (300 MHz, CDCl_3_), δ (ppm): 8.26 (s, 1H, C2--H); 7.55 (d, J = 12.0 Hz, 1H); 4.44–4.22 (m, 5H); 3.66 (s, 4H); 3.22 (s, 4H); 2.83 (s, 3H); 1.81–1.69 (m, 2H); 1.53 (d, J = 6.6 Hz, 3H); 1.39–1.16 (m, 22H); 0.87 (t, J = 6.6 Hz, 3H). ^13^C NMR (75 MHz, CDCl_3_), δ (ppm): 172.90; 165.42; 156.95; 153.76; 145.41; 140.33; 129.67; 123.9; 110.26; 105.41; 68.54; 65.29; 54.86; 54.70; 48.13; 44.36; 32.03; 29.80; 29.77; 29.68; 29.47; 28.90; 26.13; 22.80; 18.30; 14.23. HRMS (ES+) *m/z*: 558.3711 (M + 1)^+^. FT-IR (ν cm^−1^): 2918; 2849; 1718; 1618; 1578; 1544; 1481; 1452; 1412; 1319; 1248; 1182; 1090; 980; 845; 800.

Hexadecyl (S)-9-fluoro-2,3-dihydro-3-methyl-10-(4-methylpiperazin-1-yl)-7-oxo-7H-pyrido [1,2,3-de]-1,4-benzoxazine-6-carboxylate (**10**)

White solid; Yield 23%; m.p.:179–181 °C; ^1^H NMR (300 MHz, CDCl_3_), δ (ppm): 8.24 (s, 1H); 7.61 (d, J = 12.6 Hz, 1H); 4.41–4.24 (m, 5H); 3.34 (d, J = 4.2Hz, 4H); 2.55 (t, J = 4.8 Hz, 4H); 2.36 (s, 3H); 1.83–1.71 (m, 2H); 1.54 (d, J = 6.6 Hz, 3H); 1.36–1.19 (m, 26H); 0.87 (t, J = 6.6 Hz, 3H). ^13^C NMR (75 MHz, CDCl_3_), δ (ppm): 172.94; 165.97; 157.52; 154.25; 145.21; 139.69; 131.80; 123.79; 110.15; 105.89; 68.27; 65.24; 55.87; 54.83; 50.69; 46.52; 32.06; 29.83; 29.79; 29.68; 29.49; 28.94; 26.14; 22.82; 18.30; 14.23. HRMS (ES+) *m/z*: 586.4018 (M + 1)^+^. FT-IR (ν cm^−1^): 2953; 2920; 2851; 1724; 1610; 1578; 1477; 1242; 1091; 800.

Hexyl 1-cyclopropyl-6-fluoro-4-oxo-7-(piperazin-1-yl)-quinoline-3-carboxylate (**11**)

White solid; Yield 58%; m.p.:176–178 °C; ^1^H NMR (300 MHz, CDCl_3_), δ (ppm): 8.46 (s, 1H); 7.88 (d, J = 12.9 Hz, 1H); 7.31 (d, J = 6.9 Hz, 1H); 4.15 (t, J = 6.9 Hz, 2H); 3.41 (d, J = 3.8 Hz, 5H); 3.32 (d, J = 3.5 Hz, 4H); 3.22 (m, 1H); 1.63 (m, 2H); 1.28–1.20 (m, 8H); 1.00 (d, J = 3.0 Hz, 2H); 0.77 (t, J = 6.9 Hz, 3H). ^13^C NMR (75 MHz, CDCl_3_), δ (ppm): 173.90; 164.96; 155.01; 148.38; 143.39; 138.10; 123.64; 109.78; 106.08; 106.01; 64.99; 46.75; 43.29; 34.90; 31.33; 28.52; 25.54; 22.40; 13.73; 7.98. MS (ES+) *m/z*: 416.2352 (M + 1)^+^. FT-IR (ν cm^−1^): 2955; 2930; 2860; 1724; 1715; 1699; 1620; 1595; 1545; 1491; 1477; 1425; 1393; 1366; 1346; 1312; 1258; 1246; 1229; 1161; 1124; 1078; 1040; 1022; 887; 802; 619.

## Figures and Tables

**Table 1:** Results obtained from the ester degradation percentage values, after 14 days in phosphate buffer (PBS) and after 3 days in human plasma; comparison with lipophilicity (log P).

Compound	R	Degradation PBS (%)	Degradation Plasma (%)	Log P_calc_ *
LEV	H	-	-	1.79
1	C_6_H_13_	2.96	6.90	4.74
2	C_7_H_15_	1.42	10.82	5.33
3	C_8_H_17_	1.23	8.33	5.77
4	C_9_H_19_	0.55	5.06	6.13
5	C_10_H_21_	0.46	2.46	6.50
6	C_11_H_23_	0.19	2.25	6.86
7	C_12_H_25_	0	0.37	7.20
8	C_13_H_27_	0	0.35	7.54
9	C_14_H_29_	0	0.32	7.80
10	C_16_H_33_	0	0.24	8.38
CIP	H	-	-	0.96
11	C_6_H_13_	1.15	4.47	3.41

* Log P values were calculated using AlogPS 2.1. [19].

**Table 2:** Results obtained for the lethal concentration 50 (LC50), in µM, the MIC in µM and the ratio of the LC50 and MIC values for the various esters studied.

Compound	R	MIC (µM)	MBC (µM)	LC50 (µM)	MIC⁄LC50
LEV	H	0.69	1.38	335	0.002
**1**	C_6_H_13_	5.61	11.2	54.9	0.102
**2**	C_7_H_15_	5.44	10.9	23.8	0.228
**3**	C_8_H_17_	5.28	10.6	13.8	0.381
**4**	C_9_H_19_	10.2	20.5	9.71	1.06
**5**	C_10_H_21_	19.9	39.9	6.31	3.16
**6**	C_11_H_23_	38.8	77.6	12.4	3.12
**7**	C_12_H_25_	18.9	75.5	9.43	2.00
**8**	C_13_H_27_	36.8	294.3	12.2	3.02
**9**	C_14_H_29_	35.9	71.7	5.81	6.17
**10**	C_16_H_33_	273	>273	152	1.80
CIP	H	<3.77	<3.77	594	0.006
**11**	C_6_H_13_	48.1	48.1	14.9	3.23
INH	-	0.44	0.44	-	-
Puromycin	-	-	-	7.29	-

**Table 3:** Antimicrobial activity expressed as MIC and MBC values (µM) for selected Gram-negative (*E. coli*) and Gram-positive (*Staphylococcus aureus*) bacterial strains.

Compound	*Escherichia coli* ATCC 8739		Vancomycin-Resistant *Staphylococcus aureus* (VISA) CIP 106760		Methicillin-Resistant *Staphylococcus aureus* (MRSA) ATCC 43866	
	MIC	MBC	MIC	MBC	MIC	MBC
LEV	-	-	354	708	<11.1	-
**1**	35.9	71.8	574	574	144	574
**2**	69.6	69.6	278	557	139	278
**3**	135	135	67.6	135	33.8	67.6
**4**	131	131	16.4	32.8	16.4	32.8
**5**	128	510	15.9	510	15.9	31.9
**6**	248	496	31.0	496	31.0	248
**7**	242	483	242	483	15.1	483
**8**	118	471	235	471	118	471
**9**	459	459	229	459	57.4	459
**10**	437	437	218	437	109	437

## Data Availability

Data is contained within the article and Supplementary Material.

## Supplementary Materials

The following supporting information can be downloaded at https://www.mdpi.com/article/10.3390/ph15101213/s1, Table S1: Antimicrobial activity expressed as MIC and MBC values (µM) for selected Gram-positive and Gram-negative bacterial strains; Annex S1—NMR spectra used for characterization of the synthesized compounds; Annex S2—FT-IR spectra used for characterization of the synthesized compounds.
