# Supplementary material for: Fluoroquinolone Derivatives in the Treatment of Mycobacterium tuberculosis Infection
Source: Pharmaceuticals (Basel). 2022 Sep 30;15(10):1213. doi: 10.3390/ph15101213 (PMC9609866; doi:10.3390/ph15101213)
Supplement: Supplementary file 1 [file pharmaceuticals-15-01213-s001.zip › Annex 2 - FT-IR spectra.pdf]

Hexyl (S)-9-fluoro-2,3-dihydro-3-methyl-10-(4-methylpiperazin-1-yl)-7-oxo-7H-pyrido[1,2,3-de]-1,4-benzoxazine-6-carboxylate (1)

SHIMADZU

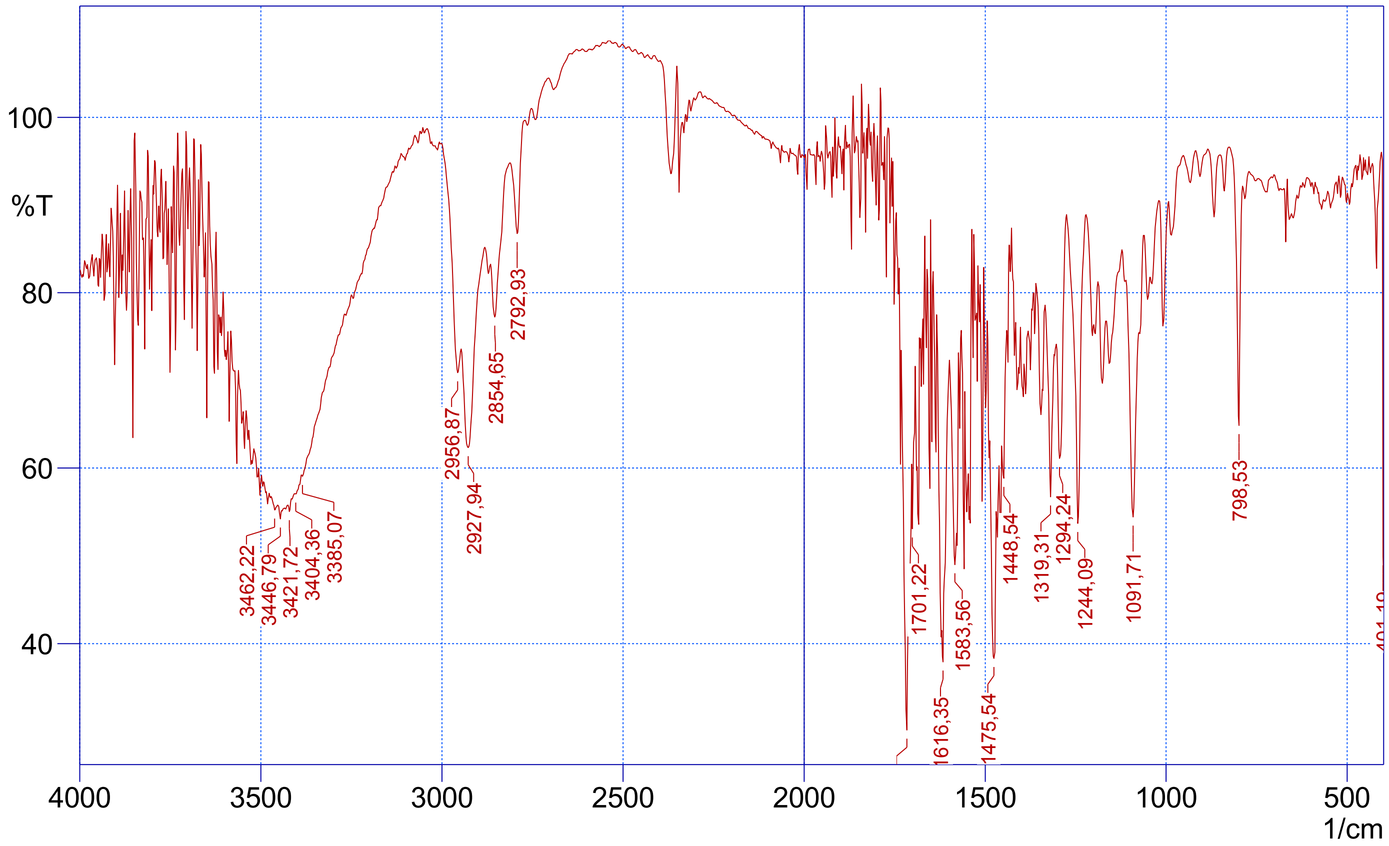

	Peak	Intensity	Corr. Intensity	Base (H)	Base (L)	Area	Corr. Area
1	401,19	47,242	23,945	403,12	399,26	1,21	0,247
2	798,53	64,879	28,444	825,53	788,89	2,424	1,498
3	1091,71	54,451	23,618	1111	1076,28	6,161	2,449
4	1244,09	53,712	35,124	1274,95	1222,87	6,468	3,793
5	1294,24	61,126	17,931	1303,88	1274,95	4,083	1,463
6	1319,31	56,727	19,629	1334,74	1303,88	5,043	1,397
7	1448,54	58,834	6,397	1452,4	1440,83	2,04	0,159
8	1475,54	38,299	19,441	1487,12	1469,76	5,994	1,952
9	1583,56	48,991	23,836	1598,99	1573,91	5,916	2,453
10	1616,35	37,925	34,05	1631,78	1600,92	8,868	4,445
11	1701,22	53,097	9,593	1703,14	1693,5	2,024	0,342
12	1716,65	30,13	30,249	1730,15	1705,07	8,868	3,289
13	2792,93	86,742	10,383	2812,21	2771,71	1,271	0,773
14	2854,65	77,226	8,491	2866,22	2812,21	3,549	0,774
15	2927,94	62,346	14,652	2947,23	2881,65	9,075	2,424
16	2956,87	70,883	6,894	3001,24	2947,23	4,279	0,331
17	3385,07	59,06	0,446	3387	3286,7	17,429	0,069
18	3404,36	57,067	0,259	3406,29	3388,93	4,125	0,051
19	3421,72	55,041	1,032	3425,58	3408,22	4,347	0,042
20	3446,79	54,214	1,395	3452,58	3437,15	3,996	0,055
21	3462,22	55,231	0,965	3469,94	3454,51	3,927	0,059

Heptyl (S)-9-fluoro-2,3-dihydro-3-methyl-10-(4-methylpiperazin-1-yl)-7-oxo-7H-pyrido[1,2,3-de]-1,4-benzoxazine-6-carboxylate (2)

SHIMADZU

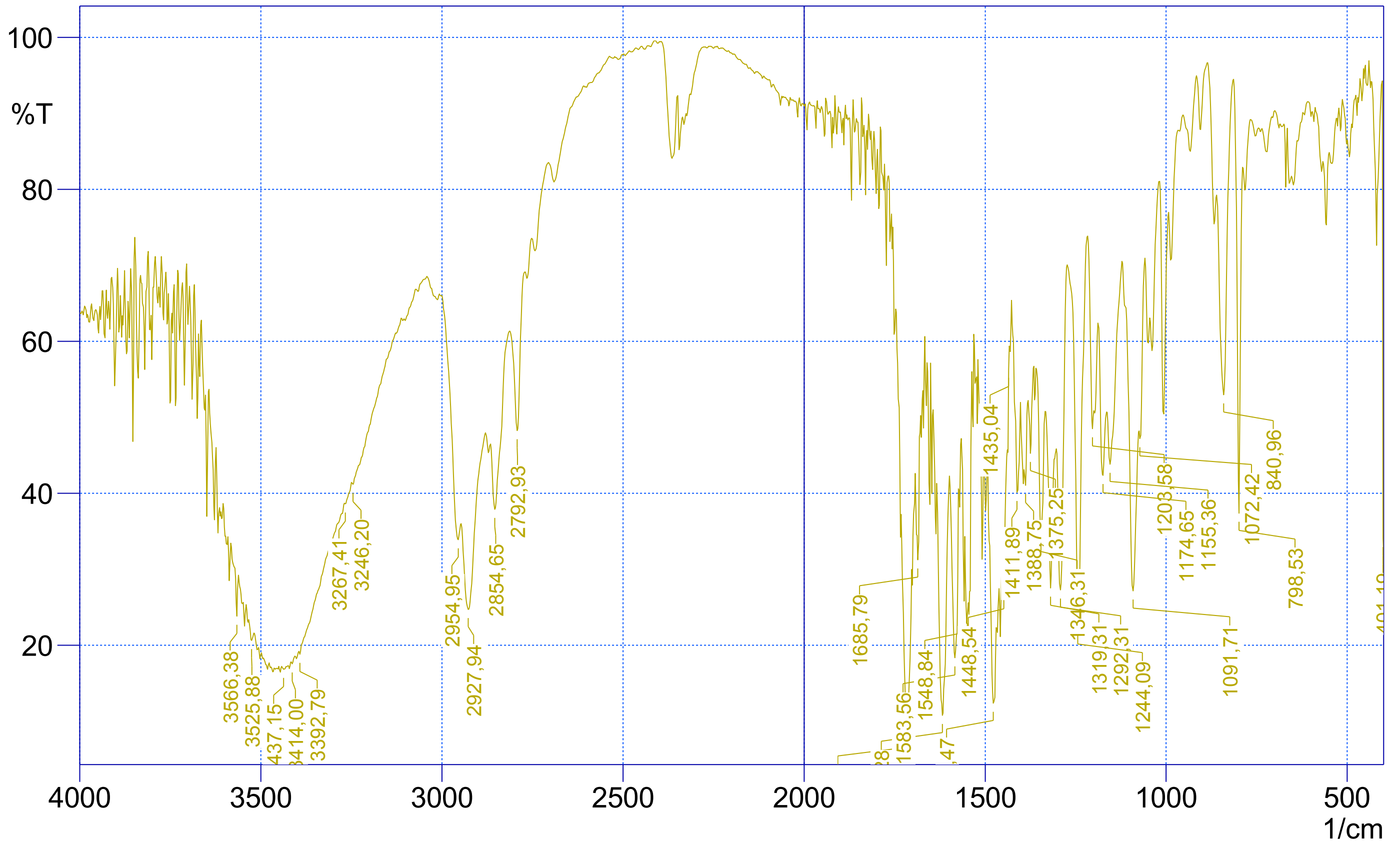

	Peak	Intensity	Corr. Intensity	Base (H)	Base (L)	Area	Corr. Area
1	401,19	31,828	31,222	403,12	399,26	1,861	0,398
2	798,53	37,38	49,969	813,96	788,89	4,486	3,1
3	840,96	52,967	32,598	860,25	813,96	6,906	3,99
4	1072,42	47,209	4,115	1074,35	1060,85	3,305	0,287
5	1091,71	27,138	28,175	1111	1076,28	13,973	5,006
6	1155,36	43,816	10,608	1163,08	1122,57	10,62	1,702
7	1174,65	42,35	13,352	1186,22	1165	6,816	1,379
8	1203,58	48,488	7,488	1217,08	1199,72	4,148	0,379
9	1244,09	22,442	49,58	1273,02	1219,01	16,034	8,307
10	1292,31	27,302	26,676	1301,95	1274,95	10,359	3,776
11	1319,31	27,536	20,908	1332,81	1303,88	11,765	2,557
12	1346,31	34,602	18,952	1357,89	1334,74	8,424	2,09
13	1375,25	45,274	8,746	1381,03	1367,53	4,045	0,496
14	1388,75	41,066	5,529	1392,61	1382,96	3,283	0,226
15	1411,89	40,138	13,908	1427,32	1404,18	7,151	1,24
16	1435,04	45,397	3,995	1436,97	1427,32	2,127	0
17	1448,54	27,087	6,472	1452,4	1440,83	5,41	0,385
18	1477,47	12,364	17,062	1492,9	1469,76	16,083	4,097
19	1548,84	24,04	13,856	1554,63	1537,27	9,243	2,649
20	1583,56	18,343	22,341	1597,06	1573,91	13,702	4,664
21	1618,28	10,766	28,666	1631,78	1598,99	22,402	9,328
22	1685,79	31,251	11,889	1691,57	1680	4,909	0,769
23	1716,65	8,833	27,841	1730,15	1693,5	25,522	10,064
24	2792,93	48,232	16,795	2812,21	2771,71	9,588	2,092
25	2854,65	37,9	11,875	2866,22	2814,14	16,499	2,493
26	2927,94	24,727	14,726	2947,23	2881,65	30,438	5,475
27	2954,95	33,908	5,458	2999,31	2949,16	16,576	1,026
28	3246,2	41,165	0,488	3248,13	3128,54	35,125	0,084
29	3267,41	38,614	0,366	3269,34	3250,05	7,719	0,08
30	3392,79	18,822	0,668	3394,72	3269,34	68,41	0,047
31	3414	17,542	0,429	3415,93	3406,29	7,162	0,039
32	3437,15	16,855	0,403	3442,94	3425,58	13,332	0,093
33	3525,88	20,621	1,619	3531,66	3520,09	7,816	0,235

Octyl (S)-9-fluoro-2,3-dihydro-3-methyl-10-(4-methylpiperazin-1-yl)-7-oxo-7H-pyrido[1,2,3-de]-1,4-benzoxazine-6-carboxylate (3)

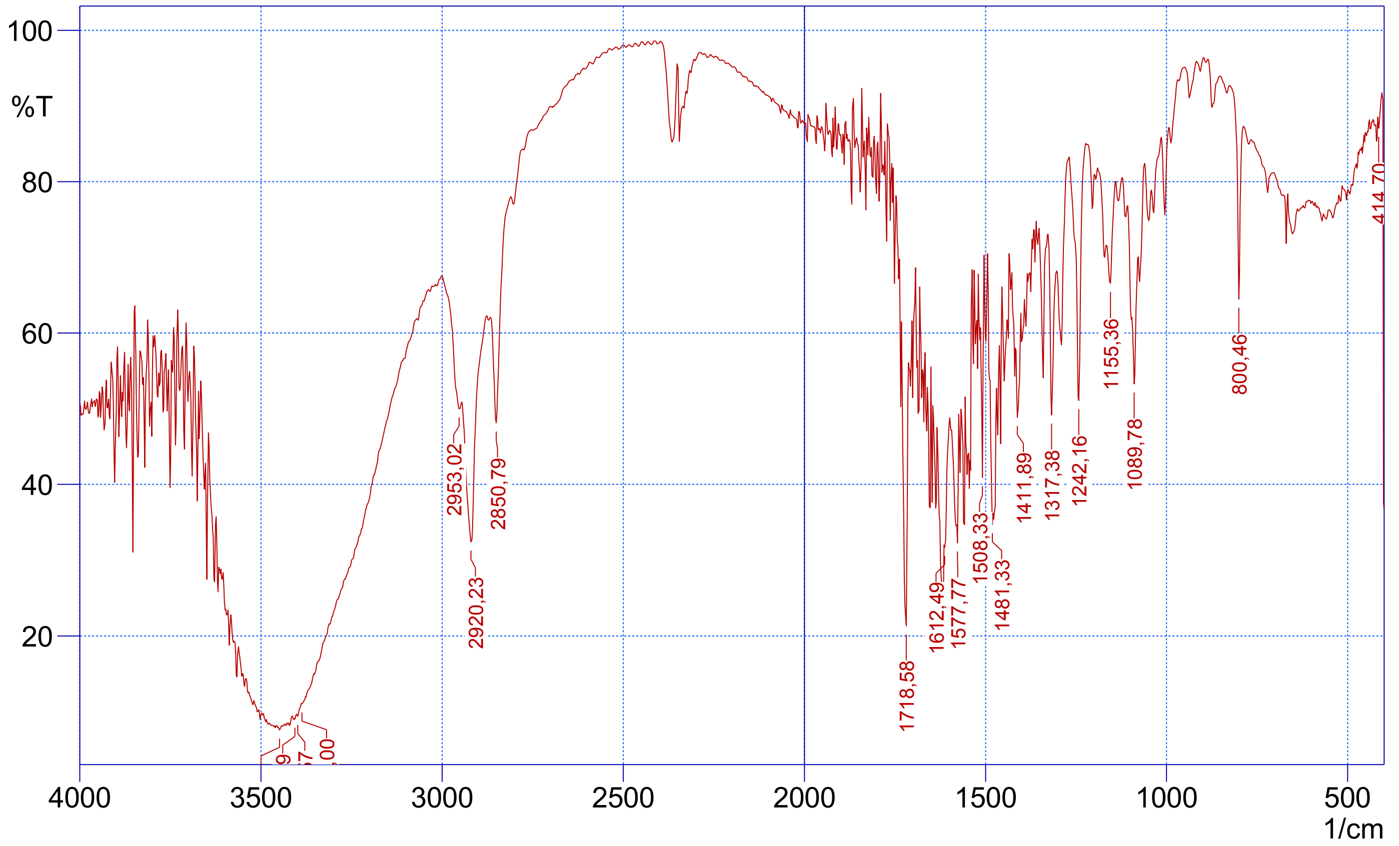

	Peak	Intensity	Corr. Intensity	Base (H)	Base (L)	Area	Corr. Area
1	414,7	87,033	2,022	416,62	405,05	0,575	0,052
2	800,46	64,465	24,732	825,53	786,96	2,866	1,095
3	1089,78	53,299	11,42	1095,57	1078,21	3,736	0,596
4	1155,36	66,549	8,93	1166,93	1141,86	3,629	0,595
5	1242,16	51,089	33,217	1269,16	1222,87	6,552	3,082
6	1317,38	49,186	21,838	1327,03	1303,88	4,962	1,43
7	1411,89	48,84	10,899	1417,68	1402,25	4,104	0,711
8	1481,33	34,68	17,163	1487,12	1471,69	6,347	1,809
9	1508,33	40,92	25,54	1514,12	1504,48	2,689	0,906
10	1577,77	32,277	16,921	1598,99	1573,91	9,804	2,049
11	1612,49	31,686	2,594	1614,42	1600,92	5,425	0,169
12	1718,58	21,336	33,956	1730,15	1712,79	8,245	3,741
13	2850,79	48,111	18,476	2866,22	2812,21	10,447	2,146
14	2920,23	32,392	22,725	2945,3	2877,79	22,178	5,419
15	2953,02	49,97	2,637	3001,24	2947,23	12,483	0,319
16	3387	11,027	0,346	3388,93	3107,32	151,959	0,055
17	3398,57	9,365	0,718	3402,43	3388,93	13,419	0,177
18	3406,29	8,997	0,607	3414	3402,43	11,979	0,191
19	3448,72	7,553	0,598	3456,44	3442,94	14,902	0,201

Nonyl (S)-9-fluoro-2,3-dihydro-3-methyl-10-(4-methylpiperazin-1-yl)-7-oxo-7H-pyrido[1,2,3-de]-1,4-benzoxazine-6-carboxylate (4)

SHIMADZU

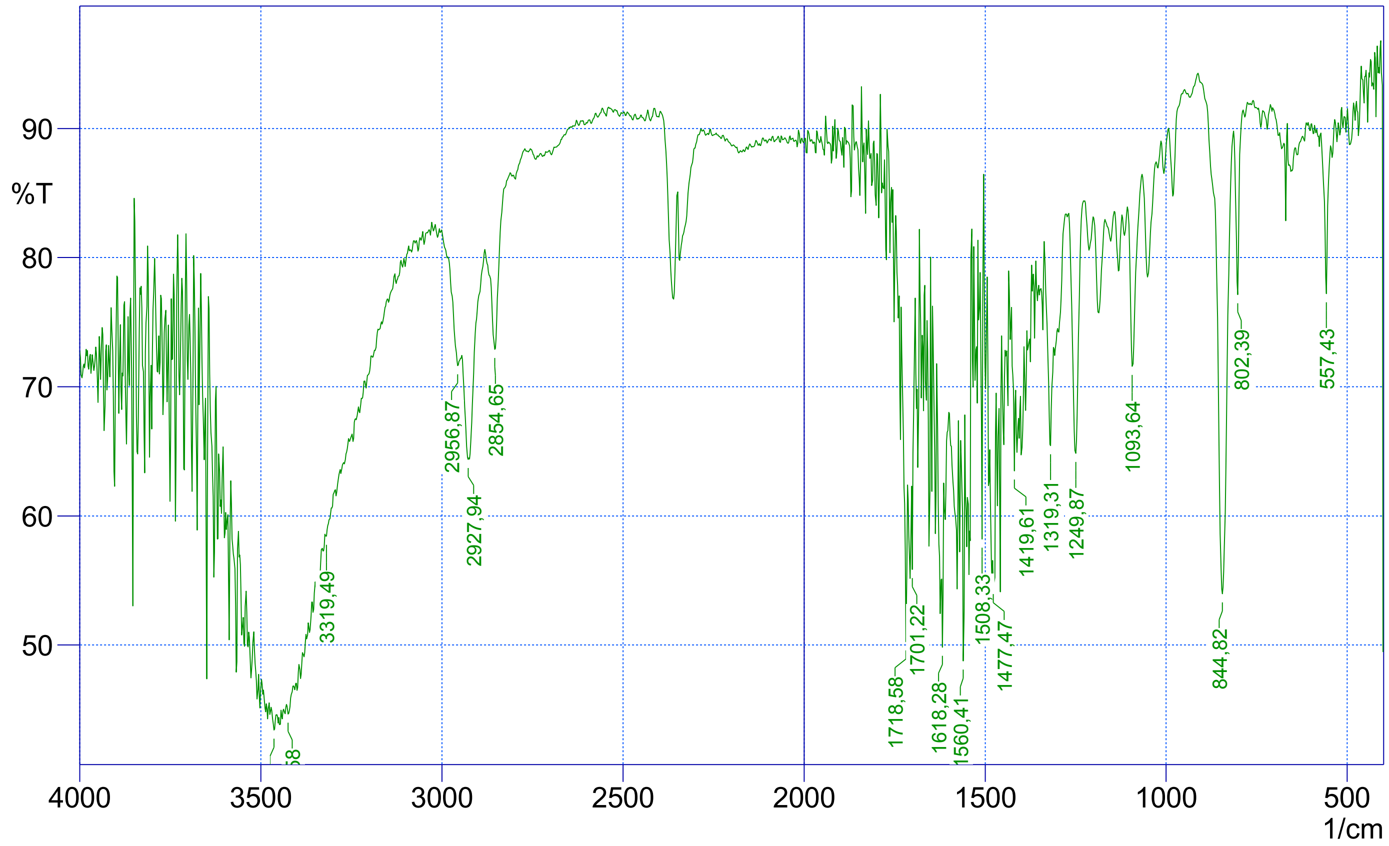

	Peak	Intensity	Corr. Intensity	Base (H)	Base (L)	Area	Corr. Area
1	557,43	77,199	11,885	572,86	545,85	1,961	0,614
2	802,39	77,141	13,358	813,96	786,96	1,793	0,637
3	844,82	53,983	36,919	912,33	815,89	9,862	6,273
4	1093,64	71,587	13,166	1107,14	1066,64	4,021	1,196
5	1249,87	64,85	19,006	1269,16	1224,8	5,342	1,97
6	1319,31	65,455	10,107	1336,67	1311,59	3,602	0,76
7	1419,61	63,478	8,383	1427,32	1415,75	1,829	0,237
8	1477,47	54,62	4,52	1479,4	1469,76	2,241	0,285
9	1508,33	58,22	25,186	1514,12	1504,48	1,435	0,633
10	1560,41	48,761	18,396	1568,13	1556,55	2,67	0,644
11	1618,28	49,84	7,744	1620,21	1614,42	1,549	0,211
12	1701,22	55,86	9,351	1703,14	1693,5	1,744	0,202
13	1718,58	50,268	15,185	1732,08	1714,72	3,566	0,761
14	2854,65	72,9	10,034	2881,65	2812,21	6,598	1,215
15	2927,94	64,367	9,872	2943,37	2883,58	8,63	1,594
16	2956,87	71,626	3,157	2981,95	2945,3	4,719	0,326
17	3319,49	58,442	0,581	3323,35	3305,99	3,925	0,035
18	3425,58	44,654	0,797	3427,51	3412,08	5,263	0,079
19	3464,15	43,417	1,333	3469,94	3458,37	4,132	0,087

Decyl (S)-9-fluoro-2,3-dihydro-3-methyl-10-(4-methylpiperazin-1-yl)-7-oxo-7H-pyrido[1,2,3-de]-1,4-benzoxazine-6-carboxylate (5)

SHIMADZU

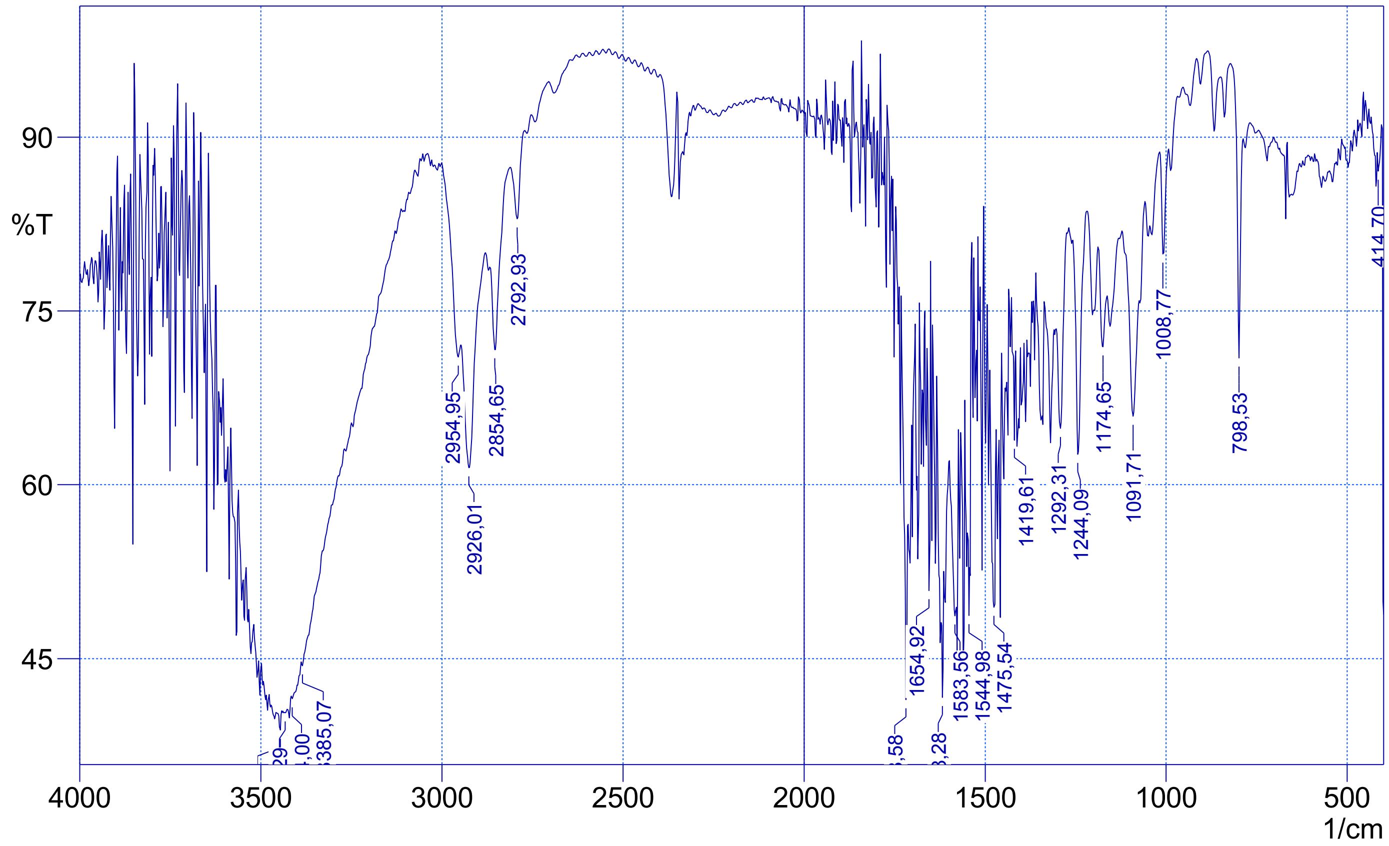

	Peak	Intensity	Corr. Intensity	Base (H)	Base (L)	Area	Corr. Area
1	414,7	87,063	1,997	416,62	405,05	0,62	0,082
2	798,53	70,949	20,947	823,6	786,96	2,059	0,91
3	1008,77	79,913	8,902	1018,41	993,34	1,77	0,481
4	1091,71	65,918	11,84	1120,64	1076,28	5,954	1,291
5	1174,65	71,896	6,345	1188,15	1163,08	3,099	0,446
6	1244,09	62,64	19,338	1257,59	1219,01	4,961	1,72
7	1292,31	64,876	11,887	1303,88	1274,95	4,142	0,981
8	1419,61	63,843	9,244	1427,32	1415,75	1,772	0,246
9	1475,54	49,429	17,032	1487,12	1469,76	4,363	1,374
10	1544,98	48,713	5,141	1548,84	1543,05	1,611	0,078
11	1583,56	48,693	3,277	1598,99	1579,7	5,193	0,246
12	1618,28	41,659	8,66	1622,13	1614,42	2,613	0,309
13	1654,92	50,875	25,396	1660,71	1651,07	2,011	0,83
14	1718,58	41,47	21,017	1732,08	1712,79	4,72	1,2
15	2792,93	82,945	5,95	2812,21	2771,71	2,548	0,489
16	2854,65	71,649	8,891	2866,22	2812,21	5,193	0,901
17	2926,01	61,473	13,14	2947,23	2881,65	10,053	2,246
18	2954,95	71,029	2,766	3001,24	2949,16	5,377	0,269
19	3385,07	44,391	0,939	3388,93	3305,99	24,297	0,191
20	3414	41,534	0,427	3415,93	3388,93	9,975	0,16
21	3433,29	40,321	0,248	3442,94	3427,51	6,072	0,024
22	3446,79	38,828	1,602	3456,44	3442,94	5,392	0,078

Undecyl (S)-9-fluoro-2,3-dihydro-3-methyl-10-(4-methylpiperazin-1-yl)-7-oxo-7H-pyrido[1,2,3-de]-1,4-benzoxazine-6-carboxylate (6)

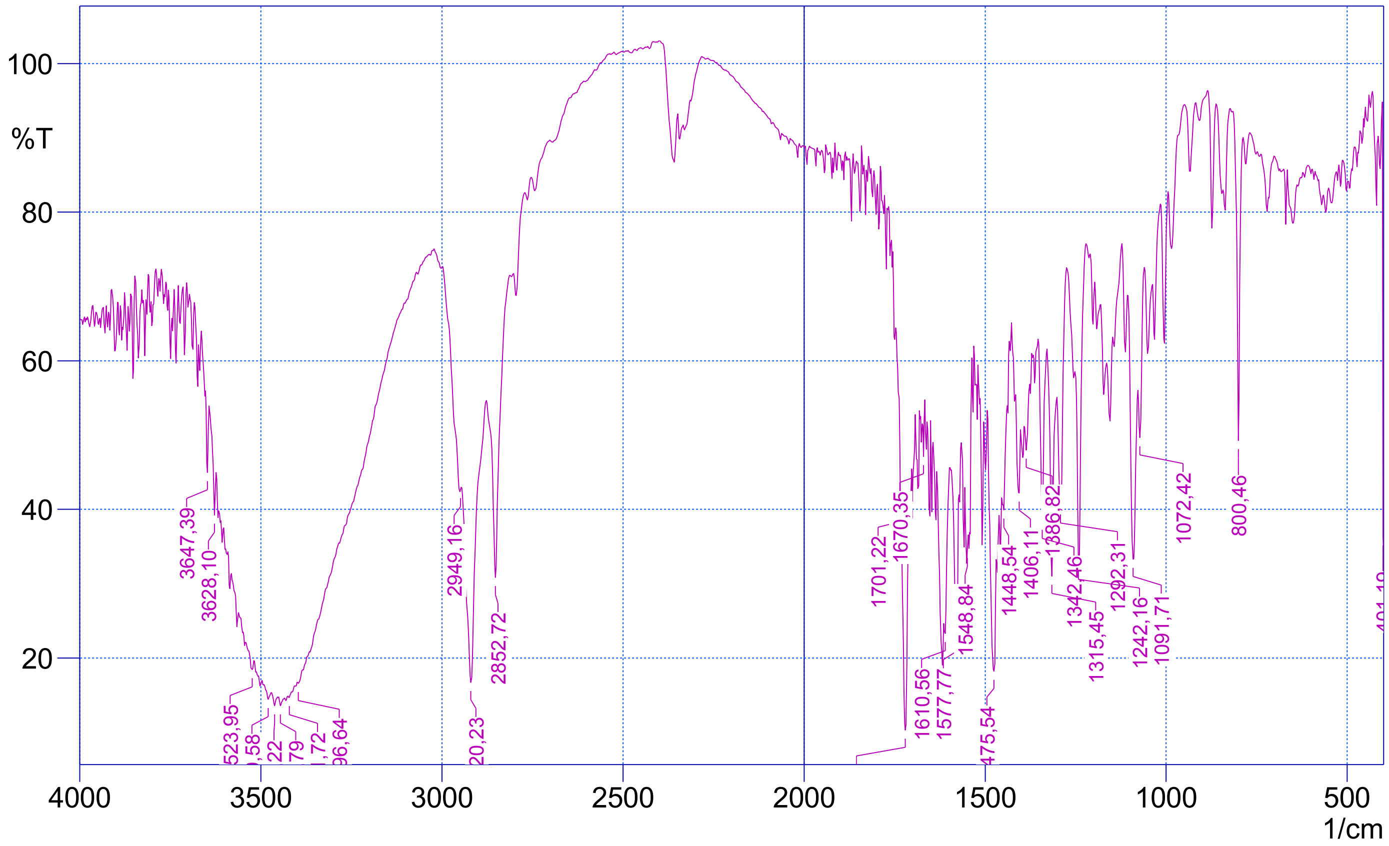

	Peak	Intensity	Corr. Intensity	Base (H)	Base (L)	Area	Corr. Area
1	401,19	33,838	30,494	403,12	399,26	1,756	0,373
2	800,46	49,272	42,131	815,89	788,89	3,146	2,106
3	1072,42	49,656	11,902	1078,21	1060,85	3,924	0,654
4	1091,71	33,321	27,952	1105,21	1080,14	8,76	3,357
5	1242,16	32,905	29,92	1249,87	1222,87	7,365	2,614
6	1292,31	40,501	20,816	1301,95	1274,95	7,267	2,02
7	1315,45	31,011	26,815	1328,95	1303,88	8,368	2,384
8	1342,46	38,428	23,284	1352,1	1330,88	6,404	1,936
9	1386,82	48,001	4,662	1390,68	1379,1	3,398	0,28
10	1406,11	42,207	9,882	1415,75	1402,25	4,554	0,798
11	1448,54	39,947	4,932	1452,4	1440,83	3,971	0,243
12	1475,54	18,221	19,785	1492,9	1469,76	13,157	4,172
13	1548,84	34,643	10,91	1554,63	1537,27	7,066	1,723
14	1577,77	23,261	18,808	1597,06	1573,91	10,823	2,407
15	1610,56	23,312	6,583	1614,42	1598,99	7,46	0,449
16	1670,35	47,141	4,999	1678,07	1668,43	2,889	0,168
17	1701,22	41,107	5,816	1703,14	1693,5	3,221	0,294
18	1720,5	10,305	40,39	1745,58	1705,07	20,609	8,988
19	2852,72	30,846	30,453	2877,79	2814,14	18,212	5,343
20	2920,23	16,743	30,554	2945,3	2879,72	30,679	10,055
21	2949,16	42,683	1,452	2997,38	2947,23	12,776	0,24
22	3396,64	16,604	0,474	3398,57	3105,39	121,785	0,046
23	3421,72	14,723	0,444	3425,58	3408,22	14,133	0,126
24	3446,79	13,597	1,084	3450,65	3437,15	11,482	0,202
25	3462,22	13,578	1,442	3469,94	3454,51	13,031	0,31
26	3479,58	14,464	1,313	3491,16	3471,87	15,778	0,334
27	3523,95	18,458	1,646	3541,31	3520,09	14,87	0,367
28	3628,1	39,234	7,564	3641,6	3624,25	5,84	0,379
29	3647,39	44,959	10,807	3664,75	3643,53	5,618	0,646

Dodecyl (S)-9-fluoro-2,3-dihydro-3-methyl-10-(4-methylpiperazin-1-yl)-7-oxo-7H-pyrido[1,2,3-de]-1,4-benzoxazine-6-carboxylate (7)

SHIMADZU

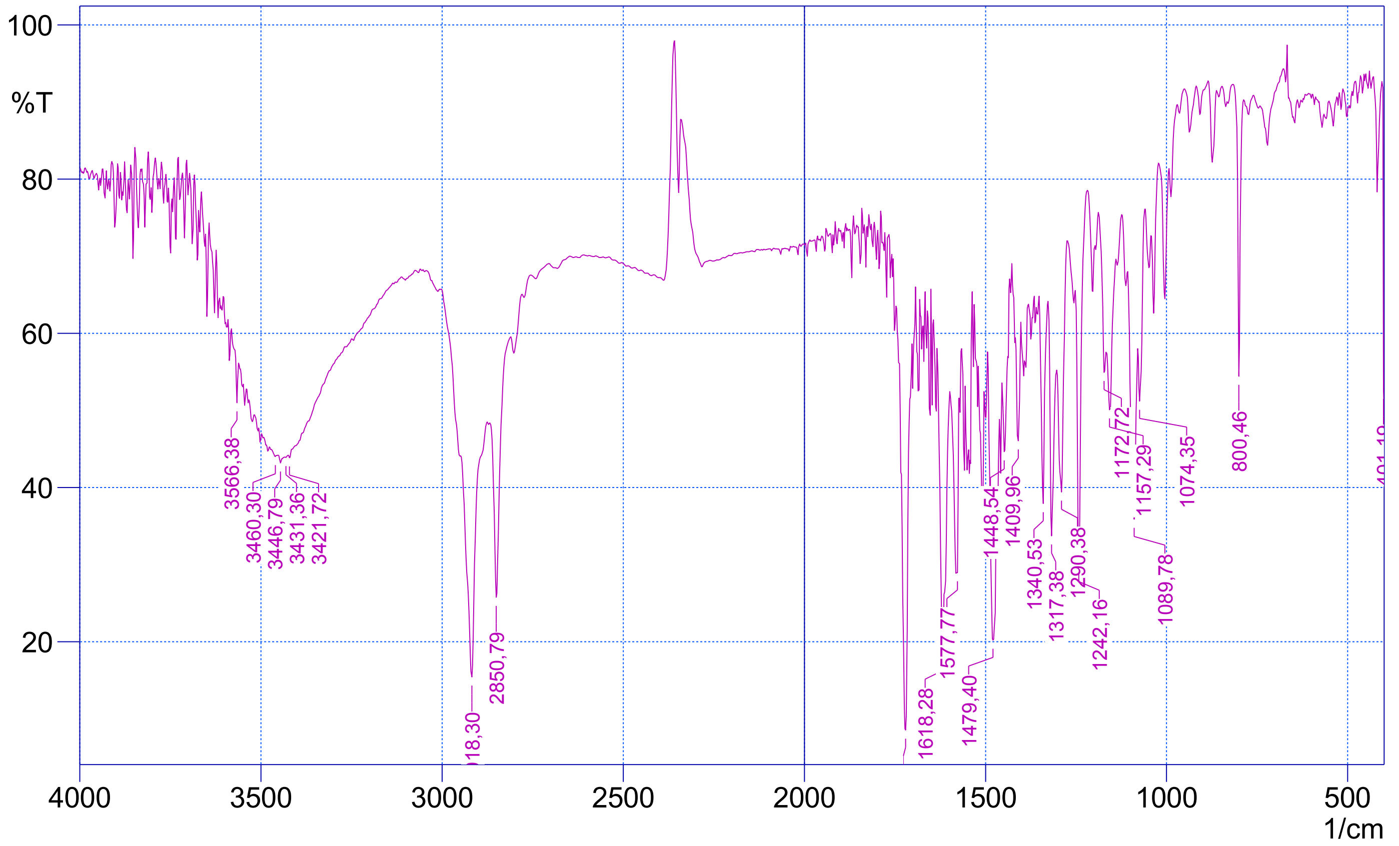

	Peak	Intensity	Corr. Intensity	Base (H)	Base (L)	Area	Corr. Area
1	401,19	49,535	21,186	403,12	399,26	1,126	0,208
2	800,46	54,452	36,551	815,89	788,89	2,645	1,549
3	1074,35	51,213	9,984	1078,21	1060,85	3,684	0,666
4	1089,78	35,902	25,696	1107,14	1080,14	8,524	3,007
5	1157,29	50,069	11,062	1165	1139,93	5,799	0,888
6	1172,72	54,919	7,916	1186,22	1166,93	3,925	0,339
7	1242,16	30,041	39,35	1251,8	1219,01	7,909	3,28
8	1290,38	39,422	22,638	1301,95	1274,95	7,682	2,328
9	1317,38	33,726	26,717	1327,03	1303,88	7,713	2,437
10	1340,53	37,919	26,336	1352,1	1328,95	6,623	2,169
11	1409,96	46,06	13,395	1415,75	1402,25	3,873	0,871
12	1448,54	44,638	7,542	1452,4	1440,83	3,655	0,496
13	1479,4	20,216	24,896	1492,9	1469,76	12,55	4,525
14	1577,77	28,94	22,684	1597,06	1573,91	9,525	2,889
15	1618,28	18,507	35,397	1631,78	1598,99	15,615	6,734
16	1720,5	8,523	49,668	1745,58	1705,07	20,188	10,855
17	2850,79	25,739	26,099	2868,15	2812,21	19,285	4,323
18	2918,3	15,422	30,528	2949,16	2877,79	33,712	9,81
19	3421,72	43,868	0,431	3423,65	3269,34	45,004	0,108
20	3431,36	43,909	0,123	3435,22	3425,58	3,437	0,006
21	3446,79	43,187	0,925	3452,58	3435,22	6,234	0,054
22	3460,3	44,031	0,479	3469,94	3452,58	6,149	0,046
23	3566,38	50,993	6,038	3581,81	3562,52	4,818	0,269

Tridecyl (S)-9-fluoro-2,3-dihydro-3-methyl-10-(4-methylpiperazin-1-yl)-7-oxo-7H-pyrido[1,2,3-de]-1,4-benzoxazine-6-carboxylate (8)

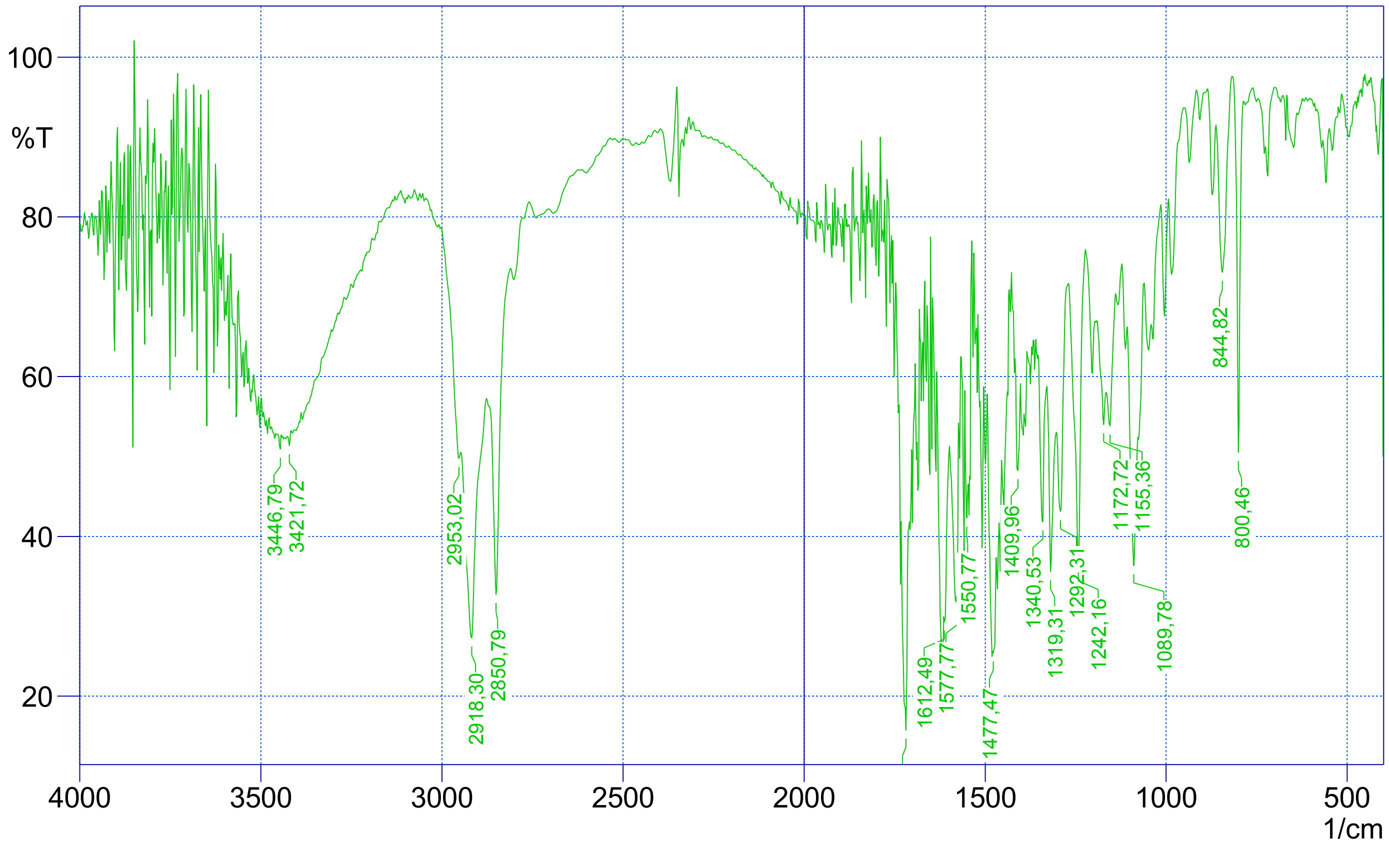

	Peak	Intensity	Corr. Intensity	Base (H)	Base (L)	Area	Corr. Area
1	800,46	50,5	45,326	817,82	786,96	2,93	2,384
2	844,82	73,08	20,81	862,18	817,82	3,386	2,296
3	1089,78	36,355	21,587	1107,14	1078,21	9,594	2,849
4	1155,36	53,858	8,907	1165	1139,93	5,613	0,735
5	1172,72	53,988	6,58	1192,01	1165	6,033	0,489
6	1242,16	36,585	37,542	1269,16	1222,87	11,26	5,16
7	1292,31	43,117	15,573	1301,95	1271,09	7,799	1,541
8	1319,31	35,562	21,004	1328,95	1303,88	8,471	2,082
9	1340,53	41,82	18,247	1352,1	1330,88	6,311	1,611
10	1409,96	48,247	12,411	1417,68	1402,25	4,202	0,849
11	1477,47	25,39	17,634	1492,9	1469,76	11,709	3,539
12	1550,77	42,31	12,295	1554,63	1539,2	4,945	1,575
13	1577,77	31,069	22,658	1598,99	1573,91	10,009	3,06
14	1612,49	29,297	3,532	1614,42	1600,92	5,624	0,3
15	1718,58	15,733	22,955	1730,15	1710,86	11,57	3,528
16	2850,79	32,723	28,857	2868,15	2812,21	14,795	4,246
17	2918,3	27,292	26,01	2947,23	2877,79	26,414	7,749
18	2953,02	49,772	2,818	3001,24	2949,16	10,304	0,247
19	3421,72	51,343	1,535	3427,51	3415,93	3,259	0,057
20	3446,79	50,91	1,766	3452,58	3444,87	2,201	0,054

Tetradecyl (S)-9-fluoro-2,3-dihydro-3-methyl-10-(4-methylpiperazin-1-yl)-7-oxo-7H-pyrido[1,2,3-de]-1,4-benzoxazine-6-carboxylate (9)

SHIMADZU

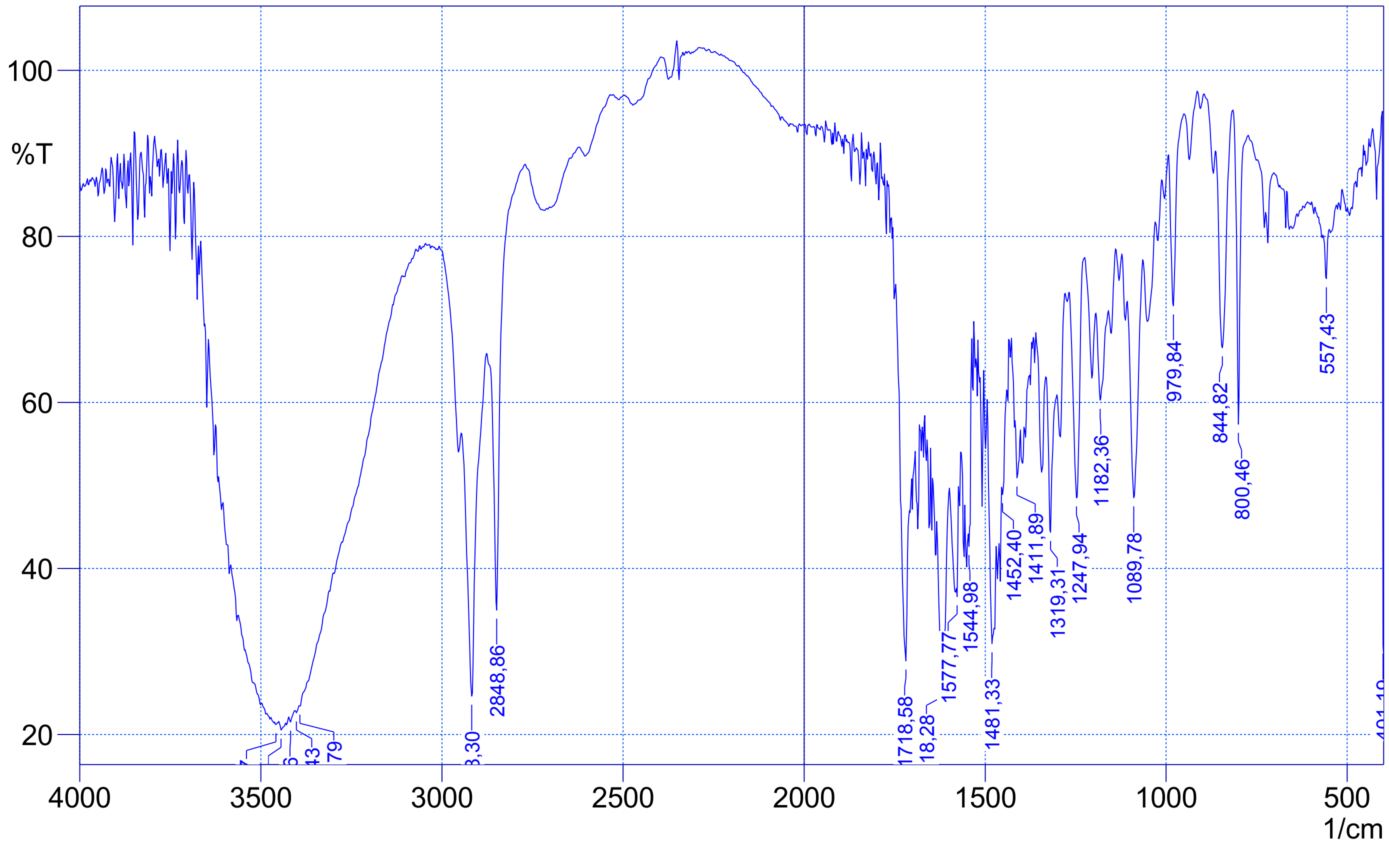

	Peak	Intensity	Corr. Intensity	Base (H)	Base (L)	Area	Corr. Area
1	401,19	29,447	32,807	403,12	399,26	2,01	0,453
2	557,43	74,911	5,623	567,07	549,71	1,853	0,217
3	800,46	57,384	36,717	815,89	773,46	3,379	2,175
4	844,82	66,608	25,641	864,11	815,89	4,526	2,94
5	979,84	71,628	19,744	993,34	950,91	2,927	1,438
6	1089,78	48,525	25,689	1107,14	1064,71	9,15	3,785
7	1182,36	60,266	10,576	1193,94	1159,22	6,328	1,141
8	1247,94	48,523	26,639	1267,23	1226,73	8,352	3,37
9	1319,31	44,381	17,589	1330,88	1303,88	7,202	1,54
10	1411,89	50,916	6,465	1417,68	1402,25	4,151	0,412
11	1452,4	48,909	2,544	1454,33	1440,83	3,544	0,171
12	1481,33	30,97	10,092	1494,83	1475,54	7,706	0,922
13	1544,98	42,671	5,897	1546,91	1539,2	2,448	0,412
14	1577,77	36,621	13,218	1598,99	1573,91	9,56	1,964
15	1618,28	26,656	18,917	1631,78	1600,92	14,237	3,844
16	1718,58	28,863	28,15	1745,58	1705,07	14,365	5,153
17	2848,86	34,988	36,758	2875,86	2771,71	15,522	4,471
18	2918,3	24,625	35,443	2945,3	2877,79	23,215	8,714
19	3392,79	23,46	0,358	3394,72	3111,18	96,749	0,02
20	3402,43	22,652	0,315	3404,36	3394,72	6,152	0,035
21	3417,86	21,518	0,88	3423,65	3406,29	11,347	0,122
22	3444,87	20,548	0,95	3450,65	3433,29	11,787	0,175
23	3458,37	21,205	0,286	3462,22	3452,58	6,469	0,026

Hexadecyl (S)-9-fluoro-2,3-dihydro-3-methyl-10-(4-methylpiperazin-1-yl)-7-oxo-7H-pyrido[1,2,3-de]-1,4-benzoxazine-6-carboxylate (10)

SHIMADZU

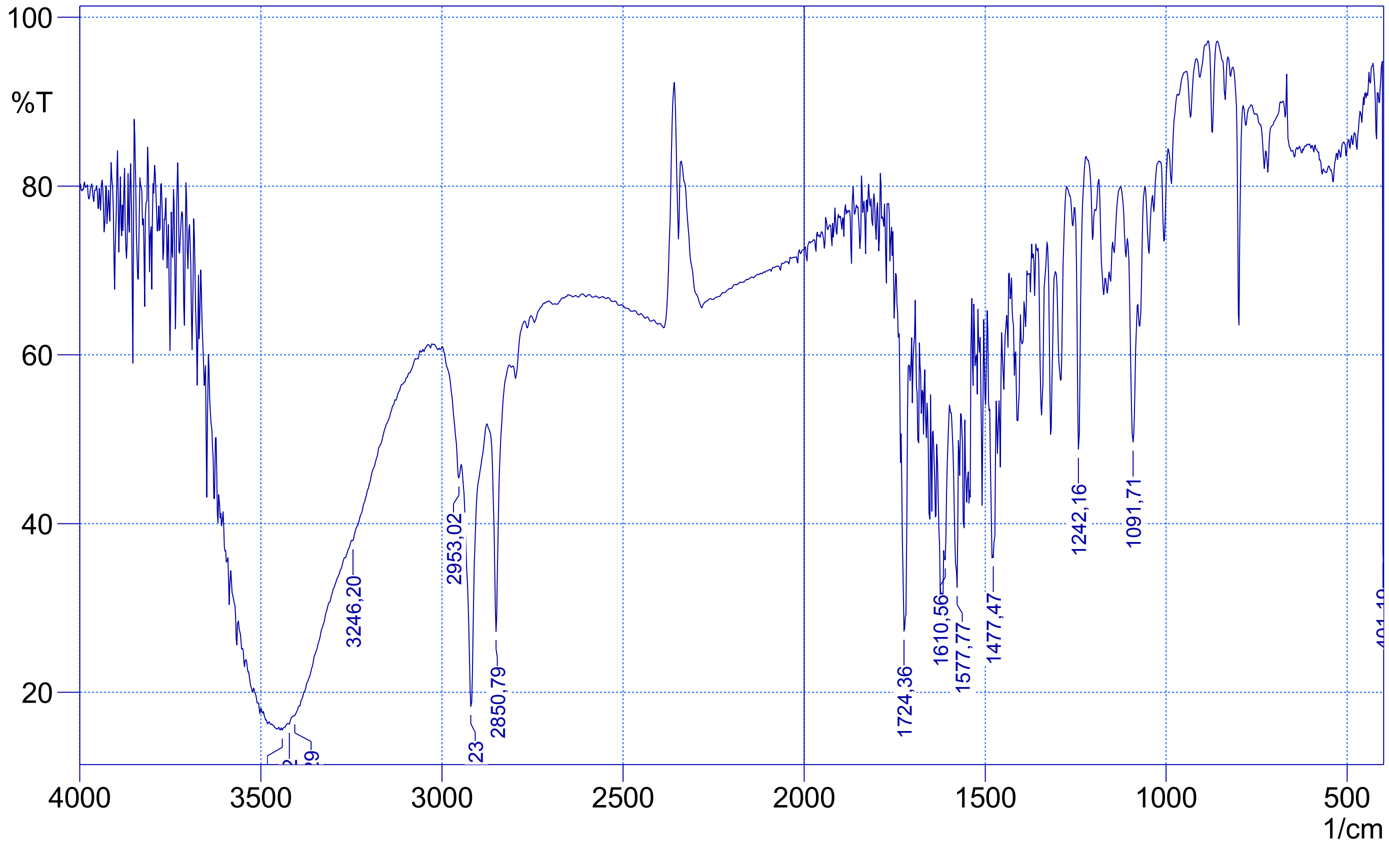

	Peak	Intensity	Corr. Intensity	Base (H)	Base (L)	Area	Corr. Area
1	401,19	34,452	30,17	403,12	399,26	1,739	0,377
2	1091,71	49,66	19,567	1105,21	1080,14	5,868	1,848
3	1242,16	48,814	30,633	1251,8	1222,87	4,785	2,072
4	1477,47	35,971	18,136	1487,12	1469,76	6,684	2,053
5	1577,77	32,45	18,016	1597,06	1573,91	8,491	1,827
6	1610,56	35,71	5,398	1614,42	1598,99	5,499	0,266
7	1724,36	27,25	35,119	1745,58	1712,79	11,604	5,118
8	2850,79	27,232	27,495	2875,86	2816,07	19,632	4,242
9	2920,23	18,345	30,264	2945,3	2877,79	28,782	8,021
10	2953,02	45,405	3,15	2999,31	2947,23	14,147	0,251
11	3246,2	38,006	0,303	3248,13	3130,47	39,416	0,018
12	3406,29	17,221	0,253	3408,22	3250,05	90,038	0,035
13	3421,72	16,219	0,244	3423,65	3412,08	8,965	0,018
14	3441,01	15,534	0,387	3444,87	3427,51	13,864	0,085

Hexyl 1-cyclopropyl-6-fluoro-4-oxo-7-(piperazin-1-yl)-quinoline-3-carboxylate (11)

SHIMADZU

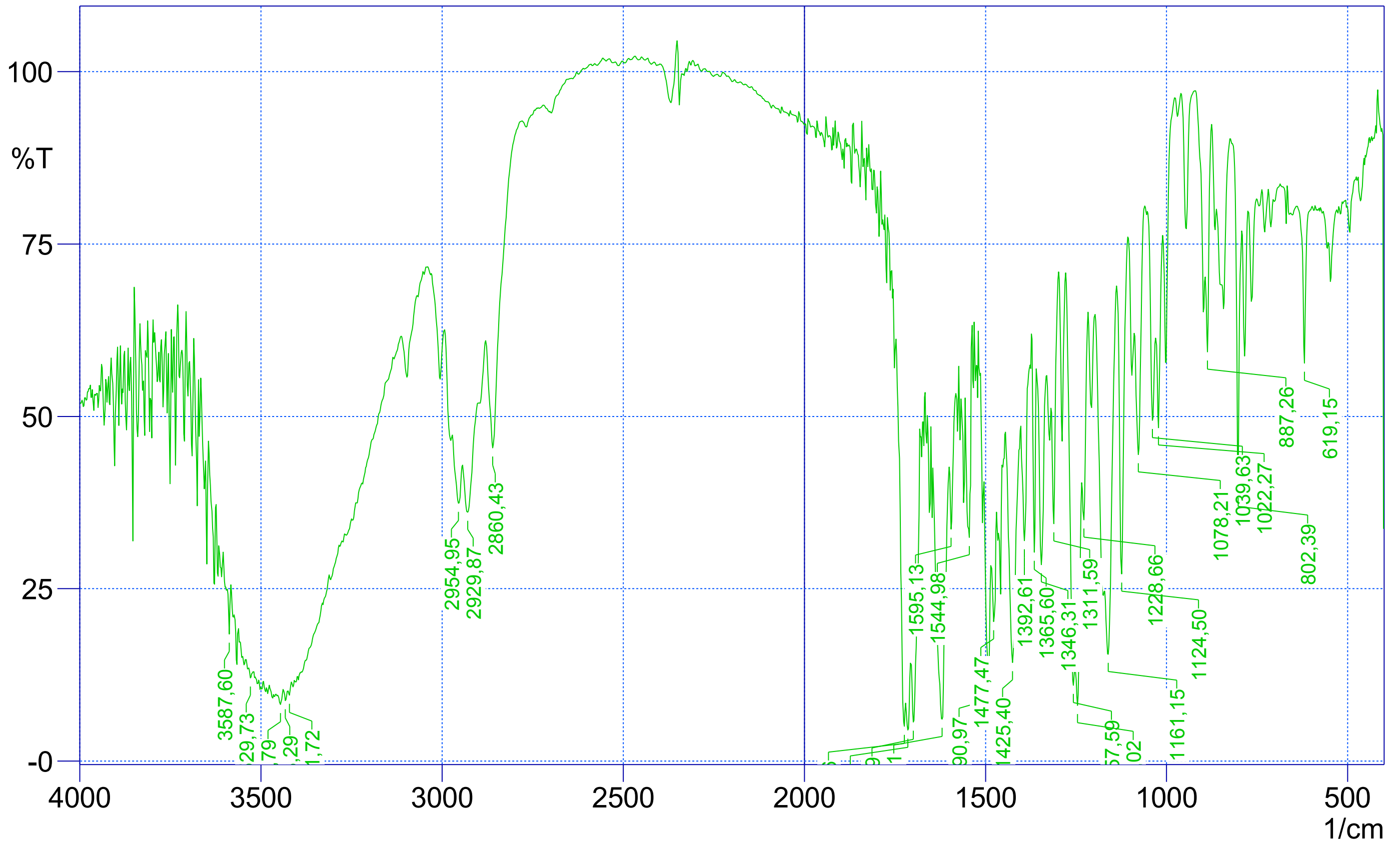

	Peak	Intensity	Corr. Intensity	Base (H)	Base (L)	Area	Corr. Area
1	619,15	57,743	22,437	638,44	603,72	4,524	1,199
2	802,39	39,453	41,619	823,6	792,74	4,592	2,166
3	887,26	59,329	18,254	893,04	875,68	2,324	0,69
4	1022,27	48,376	18,928	1029,99	1010,7	4,1	1,009
5	1039,63	49,401	18,709	1051,2	1031,92	4,299	1,123
6	1078,21	44,459	23,908	1087,85	1060,85	6,141	2,162
7	1124,5	27,166	44,007	1136,07	1107,14	8,661	4,557
8	1161,15	15,492	22,161	1170,79	1138	16,05	4,005
9	1228,66	34,992	11,241	1232,51	1219,01	4,643	0,769
10	1246,02	8,091	14,317	1251,8	1234,44	13,32	2,828
11	1257,59	11,032	11,149	1278,81	1253,73	13,838	1,265
12	1311,59	34,44	22,216	1317,38	1298,09	5,579	1,451
13	1346,31	28,5	27,278	1357,89	1332,81	9,587	3,23
14	1365,6	30,312	28,731	1371,39	1359,82	4,406	1,723
15	1392,61	31,977	20,059	1402,25	1379,1	8,455	2,055
16	1425,4	14,311	33,489	1444,68	1404,18	21,929	8,949
17	1477,47	20,222	10,362	1483,26	1471,69	7,171	1,271
18	1490,97	11,438	20,576	1504,48	1485,19	13,571	4,361
19	1544,98	32,426	22,239	1554,63	1539,2	6,331	2,115
20	1595,13	33,633	10,559	1598,99	1581,63	6,302	0,758
21	1620,21	6,071	35,157	1643,35	1600,92	31,839	15,465
22	1699,29	5,685	16,829	1705,07	1681,93	18,809	5,761
23	1714,72	4,504	6,392	1720,5	1707	15,442	2,682
24	1724,36	5,068	6,322	1747,51	1722,43	16,667	1,165
25	2860,43	45,42	21,773	2879,72	2781,35	14,915	3,735
26	2929,87	36,113	9,784	2943,37	2902,87	15,27	2,131
27	2954,95	37,412	6,954	2972,31	2945,3	10,543	1,126
28	3421,72	9,59	1,094	3425,58	3415,93	9,478	0,206
29	3433,29	8,765	1,408	3437,15	3427,51	9,917	0,336
30	3446,79	8,216	1,645	3452,58	3439,08	14,074	0,566
31	3529,73	12,094	1,236	3533,59	3522,02	10,401	0,217
32	3587,6	18,428	7,688	3603,03	3583,74	12,042	0,997
