# Supplementary material for: Fluoroquinolone Derivatives in the Treatment of Mycobacterium tuberculosis Infection
Source: Pharmaceuticals (Basel). 2022 Sep 30;15(10):1213. doi: 10.3390/ph15101213 (PMC9609866; doi:10.3390/ph15101213)
Supplement: Supplementary file 1 [file pharmaceuticals-15-01213-s001.zip › Table S1.pdf]

Table S1. Antimicrobial activity expressed as MIC and MBC values (μM) for selected Gram-positive and Gram-negative bacterial strains.

Compound	E.f. ATCC 11420		E.f. ATCC 51299		E. coli ATCC 8739		VISA CIP 106760		S.a. ATCC 6538		S.a. ATCC 43866		S.a. ATCC 700699		S.t. ATCC 13311	
	MIC	MBC	MIC	MBC	MIC	MBC	MIC	MBC	MIC	MBC	MIC	MBC	MIC	MBC	MIC	MBC
LEV	<11.07	22.14	<11.07	<11.07	-	-	354.21	708.42	<11.07	<11.07	<11.07	-	<11.07	-	<11.07	<11.07
1	287.29	574.58	287.29	287.29	35.91	71.82	574.58	574.58	143.65	287.29	143.65	574.58	143.65	-	71.82	143.65
2	278.53	557.05	139.26	278.53	69.63	69.63	278.53	557.05	139.26	139.26	139.26	278.53	139.26	-	69.63	69.63
3	67.57	135.14	67.57	135.14	135.14	135.14	67.57	135.14	67.57	67.57	33.79	67.57	67.57	-	135.14	270.28
4	32.81	16.41	32.81	32.81	131.26	131.26	16.41	32.81	16.41	32.81	16.41	32.81	-	-	65.63	262.51
5	31.90	127.59	15.95	63.79	127.59	510.35	15.95	510.35	15.95	31.90	15.95	31.90	15.95	-	255.17	510.35
6	62.06	496.47	31.03	248.24	248.24	496.47	31.03	496.47	15.51	496.47	31.03	248.24	-	-	248.24	496.47
7	120.82	241.65	15.10	120.82	241.65	483.29	241.65	483.29	30.21	483.29	15.10	483.29	120.82	-	241.65	-
8	235.42	470.83	117.71	470.83	117.71	470.83	235.42	470.83	58.85	470.83	117.71	470.83	-	-	235.42	470.83
9	229.49	458.99	114.75	458.99	458.99	458.99	229.49	458.99	57.37	458.99	57.37	458.99	229.49	-	229.49	-
10	437.00	437.00	218.50	437.00	437.00	437.00	218.50	437.00	437.00	437.00	109.25	437.00	218.50	-	452.37	-
CIP	<12.07	24.14	<12.07	<12.07	-	-	386.30	772.60	<12.07	<12.07	<12.07	-	<12.07	-	<12.07	-
11	154.03	308.06	308.06	616.11	38.51	77.01	154.03	308.06	154.03	308.06	154.03	308.06	154.03	-	77.01	154.03

*Bacterial strains used: Gram-negative bacteria: Escherichia coli (E. coli) ATCC 8739; Salmonella typhimurium ATCC 13311; Gram-positive bacteria: Enterococcus faecalis (E.f.) ATCC 11420; Enterococcus faecalis ATCC 51299; Escherichia coli (E. coli) ATCC 8739; Vancomycin-resistant Staphylococcus aureus (VISA) CIP 106760; Staphylococcus aureus (S.a.) subsp. aureus Rosenbach ATCC 6538; Staphylococcus aureus subsp. aureus Rosenbach ATCC 43866; Staphylococcus aureus CIP 106414, ATCC 700699;
